# Signatures of Rapid Evolution in Urban and Rural Transcriptomes of White-Footed Mice (*Peromyscus leucopus*) in the New York Metropolitan Area

**DOI:** 10.1371/journal.pone.0074938

**Published:** 2013-08-28

**Authors:** Stephen E. Harris, Jason Munshi-South, Craig Obergfell, Rachel O’Neill

**Affiliations:** 1 Program in Ecology, Evolutionary Biology, & Behavior, The Graduate Center, City University of New York (CUNY), New York, New York, United States of America; 2 Louis Calder Center, Fordham University, Armonk, New York, United States of America; 3 Molecular & Cell Biology, University of Connecticut, Storrs, Connecticut, United States of America; University of Massachusetts, United States of America

## Abstract

Urbanization is a major cause of ecological degradation around the world, and human settlement in large cities is accelerating. New York City (NYC) is one of the oldest and most urbanized cities in North America, but still maintains 20% vegetation cover and substantial populations of some native wildlife. The white-footed mouse, *Peromyscus* *leucopus* , is a common resident of NYC’s forest fragments and an emerging model system for examining the evolutionary consequences of urbanization. In this study, we developed transcriptomic resources for urban *P* *. leucopus* to examine evolutionary changes in protein-coding regions for an exemplar “urban adapter.” We used Roche 454 GS FLX+ high throughput sequencing to derive transcriptomes from multiple tissues from individuals across both urban and rural populations. From these data, we identified 31,015 SNPs and several candidate genes potentially experiencing positive selection in urban populations of *P* *. leucopus* . These candidate genes are involved in xenobiotic metabolism, innate immune response, demethylation activity, and other important biological phenomena in novel urban environments. This study is one of the first to report candidate genes exhibiting signatures of directional selection in divergent urban ecosystems.

## Introduction

Urbanization dramatically alters natural habitats [1], and its speed and intensity will increase as over two-thirds of the world’s human population is predicted to live in urban areas by 2050 [2]. Understanding how natural populations adapt to ecologically divergent urban habitats is thus an important and immediate goal for urban ecologists and evolutionary biologists. Few ecological and evolutionary studies are conducted in urban environments [3], but recent attitude shifts and technological advancements have removed many of the obstacles to working on urban wildlife. Multiple studies have demonstrated that urban areas are biologically diverse, productive, and viable [4], and the development of next generation sequencing (NGS) has facilitated the generation of genomic resources for uncharacterized species in natural environments [5–7]. Understanding the genetic basis of adaptation in successful urban species will aid in future conservation efforts and provide insights into the effects of other anthropogenic factors, such as global climate change and evolutionary trajectories in human-dominated environments [4,8,9].

Cities typically experience a substantial decrease in biodiversity of many taxonomic groups as urban ‘avoiders’ disappear, accompanied by a rise in urban ‘exploiters’ that are primarily non-native human commensals such as pigeons or rats. Urban ‘adapters’ are native species that favor disturbed edge habitats such as urban forest fragments, relying on a combination of wild-growing and human-derived resources [10–12]. This last group is of primary interest for examining genetic signatures of recent evolutionary change in novel urban environments. Severe habitat fragmentation is one of the primary impacts of urbanization and often leads to genetic differentiation between populations [1,13,14]. Introductions of new predators and competitors alter ecological interactions [15], and new or more abundant parasites or pathogens influence immune system processes [16]. Air, water, and soil pollution typically increase in local urban ecosystems, and selection may favor previously-rare genetic variants that more efficiently process these toxins [17–19]. Recent studies provide some evidence of local adaptation and rapid evolution in urban patches. Using a candidate gene approach, Mueller et al. [20] found consistent genetic divergence between behavioral genes for circadian behavior, harm avoidance, migratory behavior and exploratory behavior in multiple urban–rural population pairs of the common blackbird, *Turdus* *merula* . Examining phenotypes, Brady [21] found rapid adaptation to roadside breeding pond conditions in the salamander, *Ambystoma* *maculatum* , and Cheptou et al. [22] reported a heritable increase in production of non-dispersing seeds in the weed, *Crepis* *sancta* , over 5-12 generations in fragmented urban tree pits. The genetic architecture of the phenotypes under selection has not been described for either of these urban ‘adapters’, but outlier scans of transcriptome sequence datasets are one promising approach [23].

*Peromyscus* spp. are an emerging model system for examining evolution in wild populations [24–26], but large-scale genomic resources are not yet widely available. The genus contains the most widespread and abundant small mammals in North America, and *Peromyscus* research on population ecology, adaptation, aging, and disease has a long, productive history [27–31]. An increasing number of studies have demonstrated that *Peromyscus* spp. rapidly (i.e. in several hundreds to thousands of generations) adapt to divergent environments. These examples include adaptation to hypoxia in high altitude environments [26] and adaptive variation in pelage color on light-colored soil substrates [25,32,33]. Presently, *P* *. leucopus* is the sole *Peromyscus* spp. in New York City (J. Munshi-South, unpublished data) and searches of the Mammal Networked Information System (MANIS) database indicate that *P* *. maniculatus* has not occurred in NYC for several decades. In NYC, *P* *. leucopus* occupies most small patches of secondary forest, shrublands, and meadows within NYC parklands [33,34]. The smallest patches in NYC often contain the highest population densities of white-footed mice [35], most likely due to ecological release and obstacles to dispersal [36,37]. Consistently elevated population density in urban patches compared to surrounding rural populations is predicted to result in density-dependent selective pressures on traits related to immunology, intraspecific competition, and male-male competition for mating opportunities, among others [38,39].

White-footed mouse populations in NYC exhibit high levels of heterozygosity and allelic diversity at neutral loci within populations, but genetic differentiation and low migration rates between populations [40,41]. This genetic structure contrasts with weak differentiation reported for *Peromyscus* spp. at regional scales [42], or even between populations isolated on different islands for thousands of generations [43,44]. High genetic diversity within and low to nonexistent migration between most NYC populations suggests that selection can operate efficiently within these geographically isolated populations, either on standing genetic variation or *de novo* mutations. In this study we take steps to develop *P* *. leucopus* as a genomic model for adaptive change in urban environments.

Pooling mRNA from multiple individuals is an effective approach to transcriptome sequencing that avoids the prohibitive cost of sequencing individual genomes [45,46]. While pooling results in the loss of genetic information from individuals, the ability to identify SNPs in a population increases due to the inclusion of multiple individuals in the pool [47]. By analyzing SNPs within thousands of transcripts, it is feasible to identify candidate genes underlying rapid divergence of populations in novel environments [5,47–49]. Certain statistical approaches, such as the ratio between non-synonymous and synonymous (*p*_N_ ⁄ *p*_S_) substitutions, can be applied to pooled transcriptome data to identify potential signatures of selection between isolated populations [23,50,51]. If positive selection is acting on a codon, then non-synonymous mutations should be more common than under neutral expectations [52,53].

Here, we describe the results of *de novo* transcriptome sequencing, annotation, SNP discovery, and outlier scans for selection among urban and rural white-footed mouse populations. We used the 454 GS FLX+ system to sequence cDNA libraries generated from pooled mRNA samples from multiple tissues and populations. Several *de novo* transcriptome assembly programs were used and the contribution of specific tissue types to the transcriptome assembly was examined. We then identified coding region SNPs between urban and rural populations, and scanned this dataset for signatures of positive selection using *p*_N_ ⁄ *p*_S_ between populations and McDonald-Kreitman tests between multiple species. We report several candidate genes potentially experiencing directional selection in urban environments, and provide annotated transcriptome datasets for future evolutionary studies of an emerging model system.

## Results

### Sequencing and comparison of assembly methods

**Figure 1:**
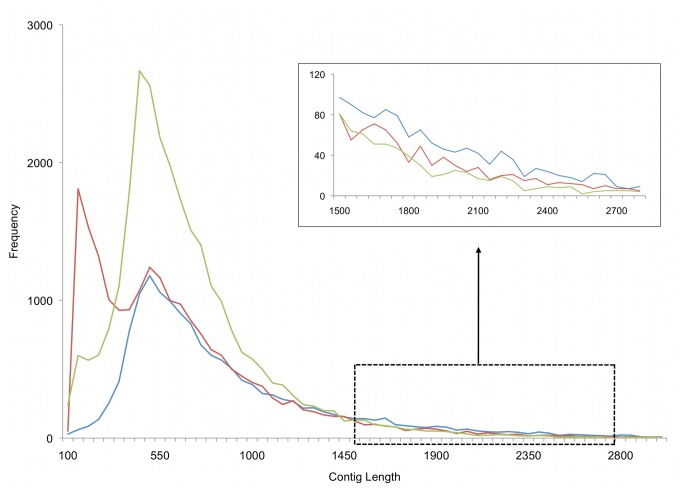
Frequency of contig lengths for three transcriptome assembly methods. Inset: Zoomed-in view of frequency of longer assembled contigs from 1,500–3,000 bp. Blue line = Newbler cDNA, Red line = Newbler genome, Green line = Cap3.

454 Sequencing of four full plates of *P* *. leucopus* cDNA libraries made from liver, brain, and gonad tissue produced 3,052,640 individual reads with an average length of 309 ± 122 bp (median = 341, Interquartile Range (IQR) = 188 bp). While the initial Newbler genomic assembly and Cap3 assembly produced more contigs, the mean length and N50 for both sets of contigs were lower than the Newbler cDNA assembly (Table 1). The Cap3 assembly and the genomic assembly included a much higher proportion of shorter contigs than the cDNA assembly (Figure 1). Coverage was calculated for all three assemblies, and all had similar median read coverage per contig (Newbler Genomic, median = 4.7 reads, IQR = 4.6; Newbler cDNA, median = 4.9 reads, IQR = 4.1; Cap3, median = 5.0 reads, IQR = 7.0, Figure S1).

**Table 1:** Results of transcriptome assembly using three different approaches.

Assembly Method	No. Contigs	Mean Contig Length (bp)	Median Contig Length (bp)	N50^*^	Length (Mb)^**^
Newbler genome^a^	20,570	630 ± 504	516	830	12.95
Cap3^b^	27,497	653 ± 380	566	732	17.95
Newbler cDNA^c^	15,004 (Isotigs)	895 ± 752	683	1,039	13.42

^a^ Newbler v. 2.5.3 large genomic assembly of total set of raw sequencing reads

^b^ Cap3 assembly using ‘assembled’ or ‘partially assembled’ reads from Newbler genome assembly

^c^ Newbler v. 2.5.3 cDNA assembly using ‘assembled’ or ‘partially assembled’ reads from Newbler genome assembly

^*^ >N50, The value where half the assembly is represented by contigs of this size or longer

^**^ Total assembly length in Megabases.

After filtering BLASTN searches against *Mus musculus* and *Rattus norvegicus* cDNA libraries, there was an average for all assemblies of 13,443 hits to known genes. The Cap3 assembly and Newbler genomic assembly produced the most hits, but the average alignment length was longest for the Newbler cDNA assembly (Table 2). Of the total number of contigs for each assembly, the Newbler cDNA assembly had the highest proportion (47%) of ‘Gene Candidates’ followed by the Cap3 assembly (42%) and the Newbler genomic assembly (41%). Assessments important for looking at *p*_N_ ⁄ *p*_S_ (longest average length of contigs, largest N50 value) and for reducing false positives (largest proportion of hits to one gene with known function) supported the assertion that Newbler’s cDNA assembly produced the best quality reference transcriptome, and all further analyses used this assembly.

**Table 2:** BLASTN search results of three *P* *. leucopus* transcriptome assemblies against reference cDNA libraries from *Mus* and *Rattus*.

Assembly Method	Total Significant Hits; *Mus*	Total Significant Hits; *Rattus*	Gene Candidates, *Mus* (^*^)	Gene Candidates, *Rattus*(^*^)
Newbler genome	12,932	12,807	8,568 (708 bp)	8,080 (714 bp)
Cap3	17,333	16,792	11,662 (623 bp)	10,938 (638 bp)
Newbler cDNA	10,699	10,094	7,048 (823 bp)	6,814 (847 bp)

^*^ Average alignment length in base pairs

Total significant hits represent sequence identity ≥ 80%, alignment length ≥ 50% of the total length of either the query or subject sequence, and *e*-value ≤ 10^-5^. Gene candidates represent significant hits where one query sequence matches one subject gene and *vice versa*.

### cDNA transcriptome assembly

The final reference *P* *. leucopus* Newbler cDNA assembly produced 17,371 contigs with an average length of 613 ± 507 bp. These contigs were assembled into 15,004 isotigs and 12,464 isogroups with a combined length of 13,421,361 bp. Isotigs were constructed from an average of 1.6 contigs and isogroups from an average of 1.2 isotigs. The contribution of sequence reads from individual tissues to the final reference transcriptome was not equal. Liver and brain cDNA libraries produced higher numbers of total reads and a greater proportion of assembled reads compared to ovary and testis libraries. The average read coverage of contigs for each tissue type varied, but coverage from liver sequences was highest with nearly 2X more compared to brain, testes, and ovaries (Table S1). Among all contigs assembled, 70% contained reads from plate 1 (normalized), 57% contained reads from plate 2 (non-normalized), 79% contained reads from plate 3 (non-normalized), and 89% contained reads from plate 4 (non-normalized). Comparison of normalized (Plate 1) and non-normalized (Plates 2-4) cDNA libraries indicated that non-normalization produced nearly twice as many total sequencing reads as compared to normalization, and non-normalized plates were able to sequence rare transcripts at a similar rate compared to the normalized plate (Table S1).

### Mouse and rat genome comparisons

**Figure 2:**
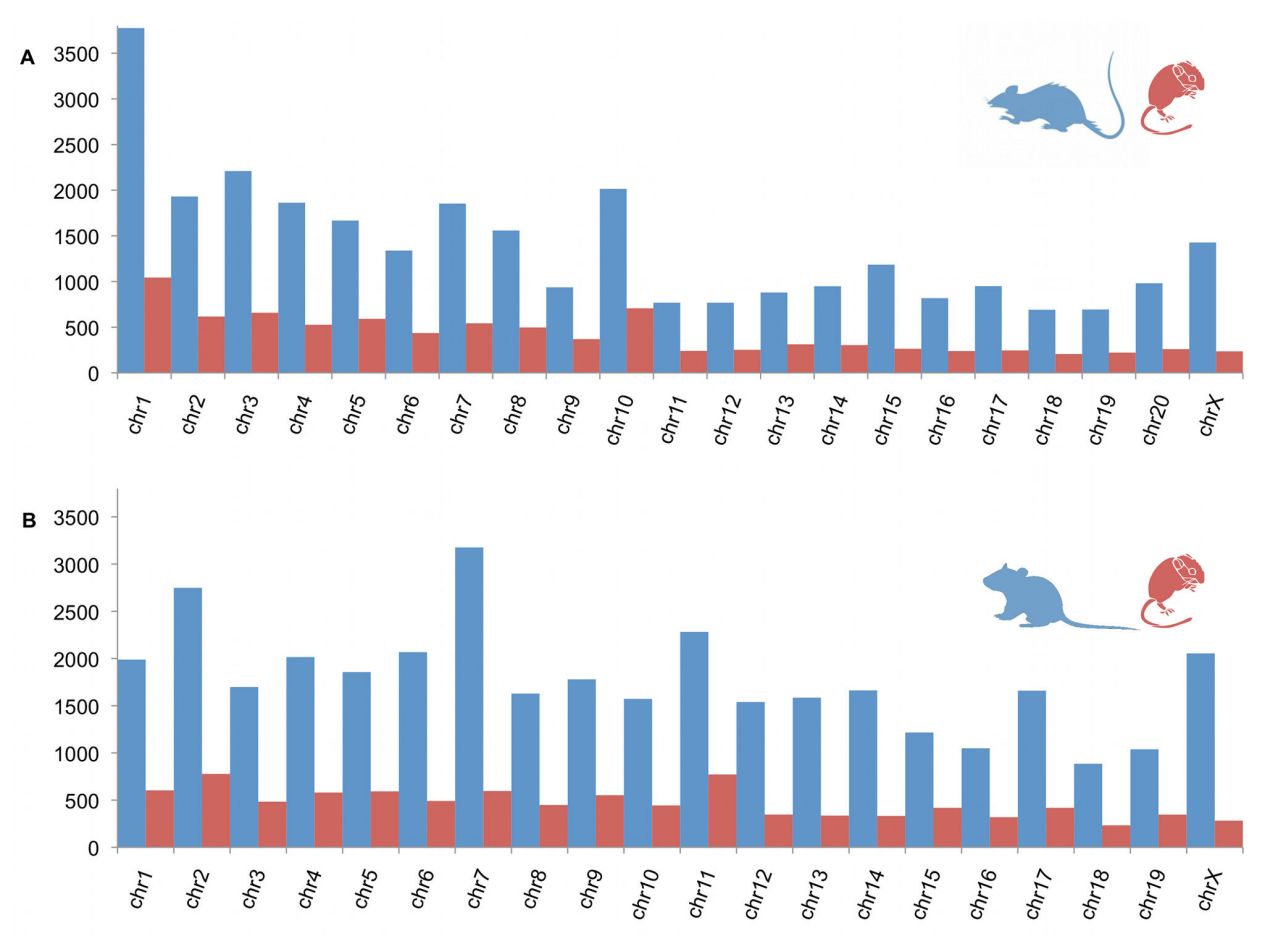
Transcriptome alignment to reference rodent genomes. Number and distribution of contigs from *P* *. leucopus* transcriptome (Newbler cDNA assembly) that aligned to each chromosome of the. (a) *Rattus norvegicus*. Blue = total number of genes per chromosome for *Rattus*. Red = number of aligned *Peromyscus* isotigs per *Rattus* chromosome. (b) *Mus musculus*. Blue = total number of genes per chromosome for *Mus* . Red = number of aligned *Peromyscus* isotigs per *Mus* chromosome.

Assembled mRNA transcripts from *P* *. leucopus* successfully mapped to both *Mus* and *Rattus* reference genomes and were distributed across all chromosomes for both references (Figure 2). There were 9,418 best BLAT hits between *P* *. leucopus* contigs and known *Mus* genes and 8,786 best hits with *Rattus* genes. The latest cDNA references include 35,900 genes for *Mus* (mm10) and 29,261 genes for *Rattus* (rn5), suggesting that full or partial coding sequence from approximately one-third to one-fourth of the *P* *. leucopus* transcriptome was sequenced. Given that many of the 15,000 contigs we assembled from our raw sequencing data may represent *Peromyscus* -specific genes not found in model rodent databases, the real proportion of the sequenced transcriptome may be much higher.

### Functional annotation

**Figure 3:**
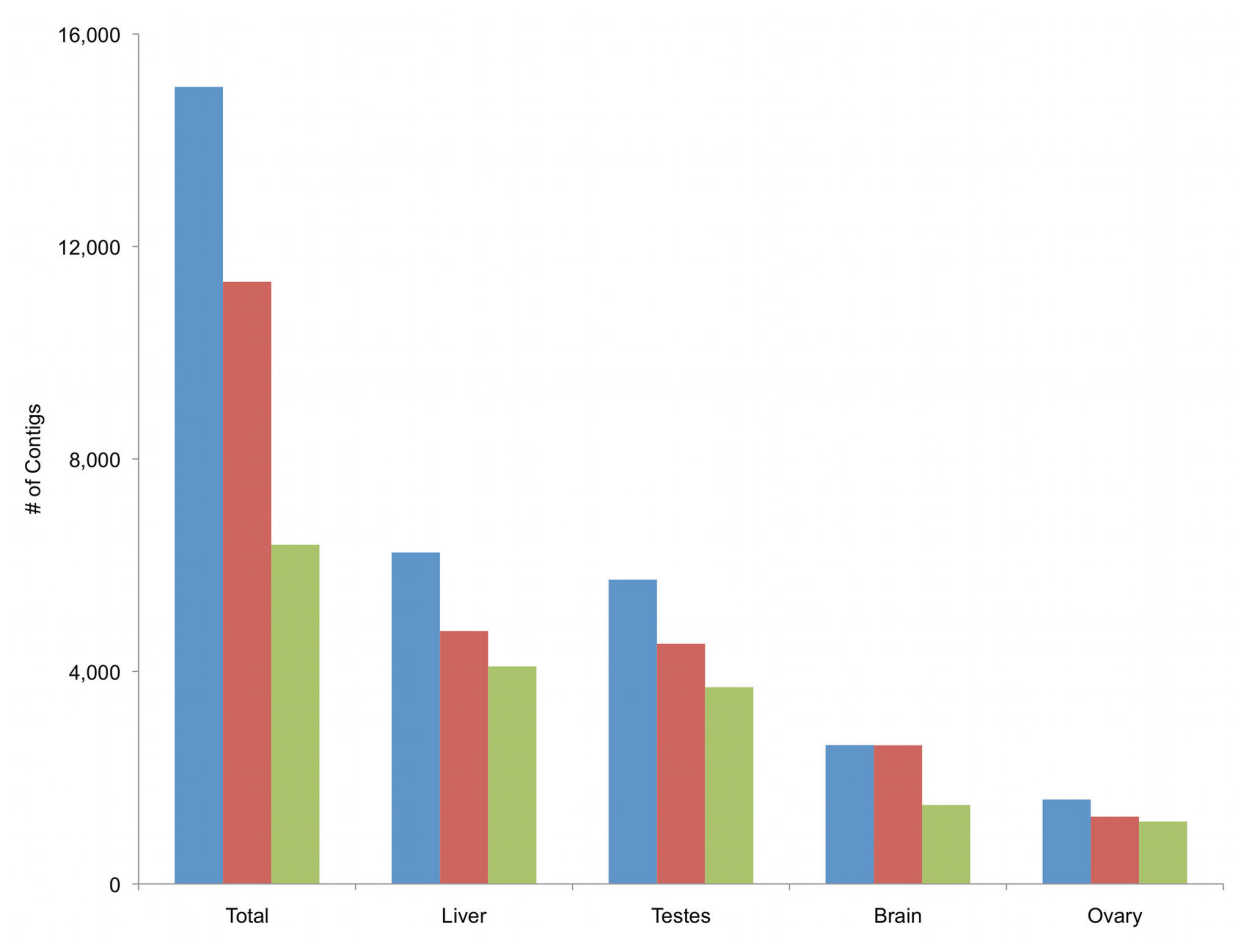
Annotation of final reference transcriptome. Number of assembled *P* *. leucopus* contigs from four different tissue types that had significant hits with known proteins on BLASTX, and GO term annotations from reference databases using Blast2Go; Blue = Total number of contigs, Red = BLASTX hits, Green = number of annotated contigs.

Among isotigs from the reference *P* *. leucopus* transcriptome, 11,355 (75.7%) had BLASTX hits to known genes, and 6,385 (42.6%) mapped to proteins and were annotated with known biological functions (GO terms) from protein databases. Top sources for these annotations were the model rodents *Cricetulus* *griseus* (3,686 BLASTX hits, 24.5%), *Mus musculus* (2,914 BLASTX hits, 19.4%), and *Rattus norvegicus* (1,671 BLASTX hits, 11.1%, Figure S2). For cDNA assemblies of individual organs, the ovary transcriptome (1,589 isotigs) had the highest proportion (73.9%) of assembled contigs with GO annotations (Figure 3). Liver (6,240 isotigs) and testes (5,728 isotigs) produced the largest number of total assembled contigs with similar proportions having GO term annotations (65.6% and 64.6%, respectively). The brain transcriptome (2,613 isotigs) included a lower number of assembled contigs and percent GO annotation (56.8%; Figure 3).

**Figure 4:**
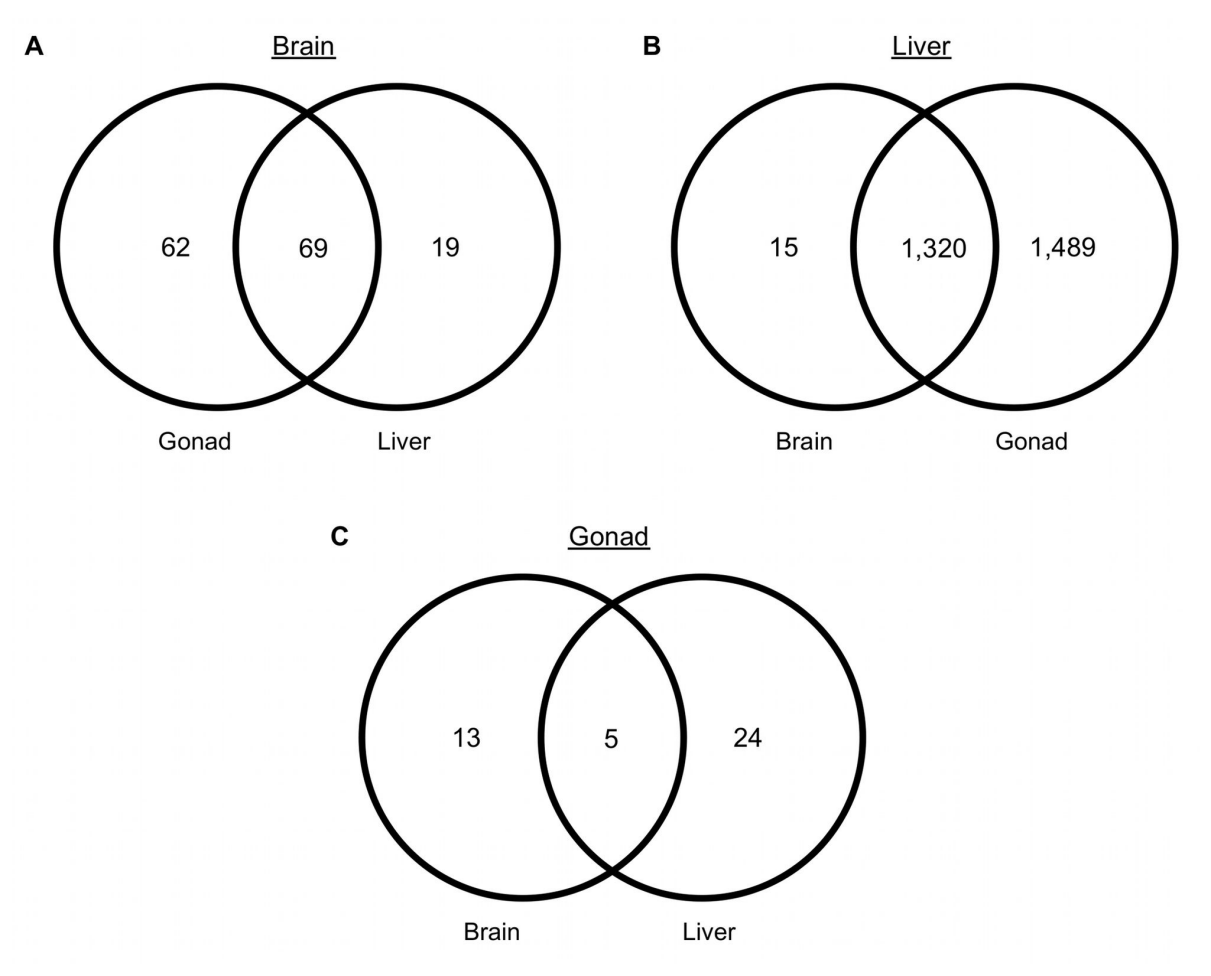
Over-represented GO terms from pairwise tissue comparisons (FDR ≤0.05). (a) Comparison of brain transcriptome to liver and gonad. (b) Comparison of liver to brain and gonad. (c) Comparison of gonad to liver and brain.

One-tailed Fisher’s Exact tests (False Discovery Rate (FDR) ≤ 0.05) indicated that liver had the most GO terms that were significantly over-represented compared to the other tissue types (Figure 4). 1,320 annotations in liver were overrepresented in both liver to brain and liver to gonad comparisons, and there were 69 overlapping annotations in brain to gonad and brain to liver comparisons (Figure 4). Gonads had the least number of annotations (five) commonly overrepresented in both brain and liver comparisons (Figure 4). When reduced to their most-specific terms, pairwise comparisons detected 64 over-represented GO annotations for liver when compared to both of the other tissues, 20 for brain, and five for gonads (Table 3). Over-represented GO terms in liver were related to metabolic processes including ATP binding, GTP binding, NADH dehydrogenase, and electron carrier activity. Over-represented GO terms in brain included regulation of behavior, actin binding, ion channel activity, motor activity, and calcium ion binding. Significantly different gonad annotations were related to reproduction, cilium (for sperm locomotion), the cell cycle, transcription regulation, and epigenetic regulation of gene expression (See Table 3 and Table S2 for full list of overrepresented GO annotations in all pairwise comparisons).

**Table 3:** Over-represented GO terms for individual tissue types from Fisher’s Exact tests (FDR ≤ 0.5) in Blast2Go.

	GO term	FDR	# Sequences
Liver			
	ATP binding	5.31E-24	184
	zinc ion binding	5.93E-20	154
	transcription factor complex	3.91E-19	148
	electron carrier activity	8.53E-18	251
	structural constituent of ribosome	5.51E-15	117
	soluble fraction	2.35E-12	97
	microsome	1.53E-10	83
	protein homodimerization activity	2.75E-10	81
	oxygen binding	1.97E-09	93
	perinuclear region of cytoplasm	9.92E-09	69
	GTP binding	7.64E-08	62
	GTPase activity	2.82E-05	42
	ubiquitin-protein ligase activity	2.82E-05	42
	NADH dehydrogenase (ubiquinone) activity	5.01E-05	40
	drug binding	6.65E-05	39
	sequence-specific DNA binding	6.65E-05	39
	double-stranded DNA binding	8.90E-05	38
	mitochondrial respiratory chain complex I	1.18E-04	37
	transcription coactivator activity	1.18E-04	37
	catalytic step 2 spliceosome	1.58E-04	36
Brain			
	protein complex	1.27E-06	569
	plasma membrane	4.30E-92	567
	signal transduction	2.15E-39	525
	cytosol	1.79E-08	411
	cell differentiation	5.07E-28	372
	anatomical structure morphogenesis	1.89E-30	291
	cell death	1.78E-06	247
	cell-cell signaling	2.79E-61	232
	ion transport	3.12E-17	209
	cytoplasmic membrane-bounded vesicle	1.33E-22	197
	golgi apparatus	1.51E-10	168
	cytoskeleton organization	9.13E-13	145
	cellular homeostasis	9.82E-16	134
	behavior	6.72E-28	133
	calcium ion binding	7.69E-13	109
	actin binding	3.54E-15	93
	response to abiotic stimulus	4.97E-08	88
	protein kinase activity	1.61E-03	77
	ion channel activity	5.21E-17	62
	motor activity	8.38E-06	48
Gonads			
	nucleic acid binding	1.87E-08	1101
	nuclear chromosome	9.86E-06	119
	reproduction	1.92E-06	680
	RNA binding	6.70E-04	637
	viral reproduction	1.74E-02	339

GO terms have been reduced to their most specific terms. Only common GO terms over represented for one tissue compared to the other two tissues are shown. The top 20 terms are shown, see Table S2 for full list of GO annotations.

### SNP calling and calculation of p_N_ ⁄ p_S_

**Figure 5:**
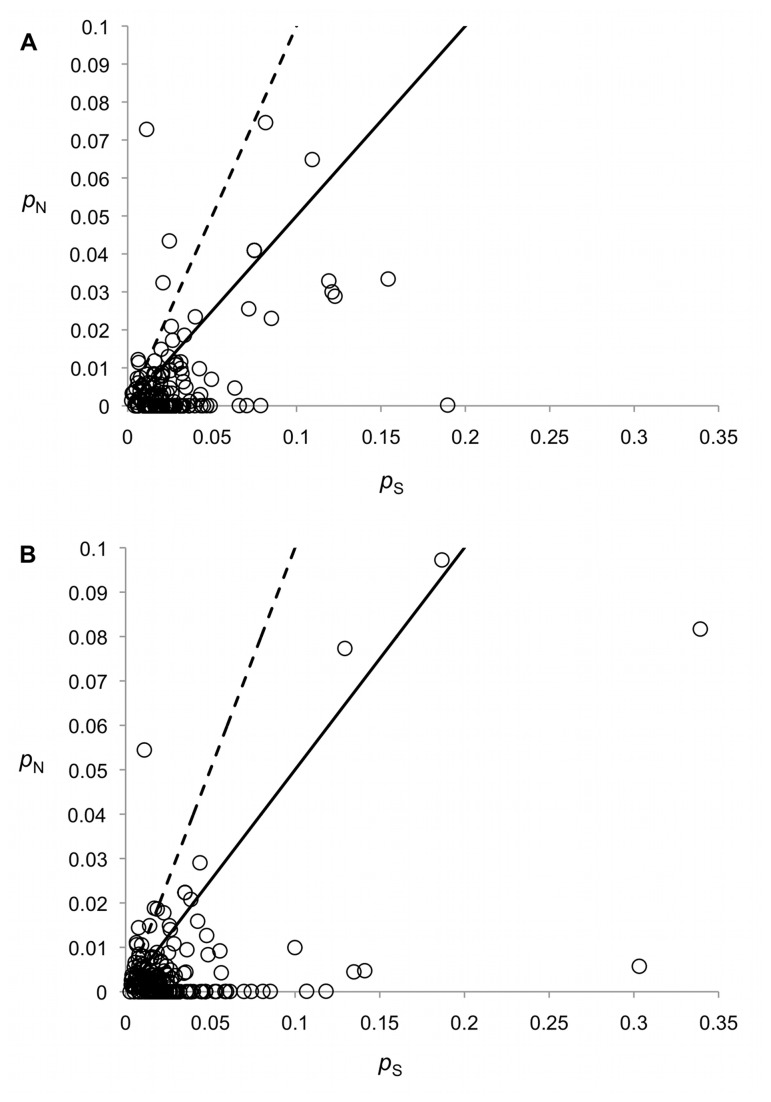
Non-synonymous (*p*_N_) SNP substitutions plotted vs. synonymous (*p*_S_) substitutions for 354 genes. Each circle represents one unique assembled contig. (a) Pairwise comparisons for all urban populations. (b) Pairwise comparisons for urban to rural populations. The dashed line denotes *p*_N_ / *p*_S_ = 1, and circles above the line (*p*_N_ / *p*_S_ > 1) indicate candidates for positive selection. The solid line shows the slope for *p*_N_ / *p*_S_ = 0.5.

After mapping the reads used in the assembly back to the Newbler cDNA reference transcriptome, 31,015 SNPs were called in 7,625 isotigs. The distribution of SNPs per isotig ranged from 1-78 (mean = 4 ± 5.4; median = 2). ORFs were identified in 11,704 isotigs comprising 5.6 Mb of sequence, and 2,655 putative ORFs contained 4,893 SNPs. Of these SNPs, 1,795 (36.6%) were classified as non-synonymous and 3,098 (63.3%) were classified as synonymous. Aligned ORFs were used to calculate *p*_N_ ⁄ *p*_S_ between each pair of populations. The majority of the ORFs did not exhibit statistical signatures of positive selection (overall mean ± SE *p*_N_ ⁄ *p*_S_ = 0.28 ± 0.56). For the 2,307 pairs of homologous cDNA sequences between populations that contained predicted ORFs, did not contain in-frame stop codons, and had greater than or equal to three SNPs, *p*_N_ ⁄ *p*_S_ values for 11 (0.5%) contigs exceeded 1.0 (Table 4, Figure 5). The proportion of genes with *p*_N_ ⁄ *p*_S_ > 1.0 is comparable to similar studies; Sun et al. [23] found that 0.4% of genes in their *Pomaceacanaliculata* dataset were positively selected, Renaut et al. [54] reported 0.5% in *Coregonus* *clupeaformis* , and Wang et al [55] reported 1.8% in *Bemisia* *tabaci* . Four contigs (0.2%) exhibited *p*_N_ ⁄ *p*_S_ values > 1.0 in urban to urban comparisons and 7 contigs (0.3%) in urban to rural population comparisons. 42 (1.8%) contigs were found with *p*_N_ ⁄ *p*_S_ between 0.5 and 1 (Table S3, Figure 5); *p*_N_ ⁄ *p*_S_ greater than 0.5 is a less conservative filter for detecting positive selection, especially when using truncated ORFs [56,57].

**Table 4:** Candidate loci exhibiting *p*_N_ ⁄ *p*_S_ > 1.

	Sequence name	*p*_*N*_ * ⁄ p*_*S*_	Gene name	Gene function
Pairwise Urban:Rural Comparisons				
	HP_contig01773	1.01	Translocation protein SEC62	Post-translational protein translocation into the endoplasmic reticulum; plasma membrane protein
	HP_contig02632	1.05	39S ribosomal protein L51	Part of mitochondrial ribosomal large subunit (39S); involved in protein translation
	HP_contig02656	1.07	Histone H1-like protein in spermatids 1	Transcriptional regulation and / or chromatin remodeling through DNA binding during spermatogenesis
	HP_contig01778	1.12	PHD finger protein 8	Removal of methyl groups from histones
	HP_contig01919	1.18	Aldo-keto reductase family 1, member C12	Xenobiotic metabolism; oxidation-reduction process
	HP_contig00870	1.74	Camello-like 1	Metabolic process; mitochondrial inner membrane protein
	HP_contig01783	1.89	Cytochrome P450 2A15	Metabolic process; testosterone 7a-hydroxylase activity
Pairwise Urban:Urban Comparisons				
	CP_contig00473	1.23	Fibrinogen alpha chain	Glycoprotein circulating in the blood; functions in blood coagulation and part of the most abundant component of blood clots
	CP_contig01204	1.55	Solute carrier organic anion transporter family member 1A5	Membrane protein; transports hormones; facilitates intestinal absorption of bile acids and renal uptake of indoxyl sulfate
	CP_contig00256	1.76	Serine protease inhibitor a3c	Bind to proteases and inhibit proteolysis; often involved in blood coagulation and inflammation
	CP_contig00748	1.97	Alpha-1-acid glycoprotein 1	Transport protein in the bloodstream; binds and distributes synthetic drugs throughout body; modulates innate immune response

Different genes showed strong (*p*_N_ ⁄ *p*_S_ > 1) signatures of selection when urban populations were compared to other urban populations than when urban and rural populations were compared. Candidate genes identified from the ORF pairs (i.e. *p*_N_ ⁄ *p*_S_ > 1) in urban to rural comparisons were related to metabolic processes (including xenobiotic metabolism), reproduction, and demethylation (Table 4). Three genes were involved in metabolic processes: *cytochrome P450 2A15* (xenobiotic metabolism, HP_contig01783, *p*_N_ ⁄ *p*_S_ = 1.89), *camello-like 1* (HP_contig00870, *p*_N_ ⁄ *p*_S_ = 1.74), and *aldo-keto reductase* family *1, member C12* (Xenobiotic metabolism, HP_contig01919, *p*_N_ ⁄ *p*_S_ = 1.18). Our analysis also identified a reproductive gene, *histone H1-like protein in spermatids 1* (HP_contig02656, *p*_N_ ⁄ *p*_S_ = 1.07) that is involved in transcriptional regulation during spermatogenesis. The gene *phd finger* protein *8* (HP_contig01778, *p*_N_ ⁄ *p*_S_ = 1.12), codes for a demethylase that removes methyl groups from histones.

Candidate genes in urban to urban population comparisons were primarily involved in immune system processes. One of these genes is involved in regulating the innate immune response, *alpha-1-acid glycoprotein 1* (CP_contig00748, *p*_N_ ⁄ *p*_S_ = 1.97), by modulating innate immune response while circulating in the blood. The other immune system genes are involved in blood coagulation and inflammation, *serine protease inhibitor a3c* (CP_contig00256, *p*_N_ ⁄ *p*_S_ = 1.76) and *fibrinogen alpha chain* (CP_contig00473, *p*_N_ ⁄ *p*_S_ = 1.23). We also identified *solute carrier organic anion transporter family* member *1A5* (CP_contig01204, *p*_N_ ⁄ *p*_S_ = 1.55), a gene that facilitates intestinal absorption of bile acids and renal uptake and excretion of uremic toxins.

For the 22 contigs with *p*_N_ ⁄ *p*_S_ between 0.5 and 1 for urban to rural comparisons, genes are primarily involved in the innate immune response, metabolic processes, and methylation activity, and some of these genes are involved in the same biological pathways as genes listed above that exhibited *p*_N_ ⁄ *p*_S_ > 1 (Table 4, S3). For the 20 contigs with *p*_N_ ⁄ *p*_S_ between 0.5 and 1 for urban pairwise comparisons, genes are primarily involved with the innate immune response, metabolic processes (including xenobiotic), and reproductive processes.

Candidate genes were scanned for evidence of recombination using a phylogenetic framework. The Genetic Algorithm Recombination Detection (GARD) analysis identified no evidence of recombination in any potential candidate genes. Would-be breakpoints were identified in the genes *Translocation protein SEC62, Histone H1-like protein in spermatids 1, Aldo-keto reductase family 1 member C12, Fibrinogen alpha chain, Solute carrier organic anion transporter family* member *1A5*, and *Serine protease inhibitor a3c*, but Kishino-Hasegawa testing implemented in the Data Monkey web server found the signal most likely resulted from evolutionary rate variation as opposed to recombination.

McDonald-Kreitman tests were then performed to examine potentially adaptive evolution between species in all the identified candidate genes. *P* *. leucopus* was compared to *R. norvegicus*, and *C* *. griseus* when *Rattus* sequences were not available. This approach minimized the number of multiple mutations at individual sites, but results were very similar when the orthologous candidate genes were compared to any rodent with available orthologous gene sequence. Excess adaptive change (diversifying selection between species) was not identified in any of the candidate genes. For four genes, *39S ribosomal protein L51, PHD finger* protein *8, Cytochrome P450 2A15*, and *Solute carrier organic anion transporter 1A*, the ratio of non-synonymous to synonymous polymorphisms within *P* *. leucopus* was significantly higher than the ratio for divergent genetic changes between species (Table 5). While there were more non-synonymous polymorphisms than synonymous polymorphisms in the remaining seven genes, results were not significantly different from expected neutral evolution.

**Table 5:** McDonald-Kreitman tests for candidate genes with *p*_N_ ⁄ *p*_S_ > 1.

	Polymorphisms			Divergence				
Gene Name	Non-synonymous (*P*n)	Synonymous (*P*s)	Ratio (*P*n/*P*s)	Non-synonymous (*D*n)	Synonymous (*D*s)	Ratio (*D*n/*D*s)	Neutrality Index	P-value
Translocation protein SEC62	2	1	2	18	28	0.64	3.11	0.55
39S ribosomal protein L51	4	1	4	15	32	0.47	8.53	0.05
Histone H1-like protein in spermatids 1	2	1	2	20	12	1.67	1.20	1.00
PHD finger protein 8	9	3	3	36	51	0.71	4.25	0.03
Aldo-keto reductase family 1, member C12	3	1	3	18	37	0.49	2.67	0.08
Camello-like 1	4	1	4	41	23	1.78	2.24	0.65
Cytochrome P450 2A15^*^	6	1	6	13	28	0.46	12.92	0.01
Fibrinogen alpha chain	3	1	3	101	93	1.08	2.76	0.62
Solute carrier organic anion transporter 1A5	9	3	3	21	37	0.57	5.29	0.02
Serine protease inhibitor a3c^*^	4	1	4	25	19	1.32	1.27	0.65
Alpha-1-acid glycoprotein 1	4	1	4	68	44	1.55	2.59	0.65

Comparison of the amount of polymorphisms in candidate ORFs to that of the divergence in orthologous genes between *Peromyscus* and *Rattus norvegicus*. P-values were generated from Fisher’s Exact Test.

^*^ McDonald Kreitman test used *Cricetulus* *griseus*

## Discussion

### De novo transcriptome assembly and characterization

Compared to other NGS technologies, 454 transcriptome sequencing provides longer read lengths ideal for *de novo* assembly [58] and is especially useful for organisms without extensive genomic resources like *P* *. leucopus* [51,54,59–61]. We compared the relative merits of two established long-read assembly programs, CAP3 and Newbler, for assembling our transcriptomes [60,61]. Despite the substantially fewer megabases per run generated by 454 FLX+ compared to Illumina or SOLiD sequencing [62], we still ran into computational limitations during assembly when using options for cDNA sequence. Similar to Cahais et al. [63], we had the most success after compressing the raw reads into a smaller number of partially assembled sequences using a genome assembler followed by another assembly method better suited for transcriptome data. While the CAP3 assembly produced more contigs, the Newbler v. 2.5.3 transcriptome assembly performed better based on assessments useful for downstream population genomic analyses (e.g. number of long contigs and average contig length). Newbler performed well at assembling full-length cDNA contigs, and our results are in line with Mundry et al.’s [64] findings that Newbler outperformed other assembly programs in simulated experiments. The N50 value reported here is comparable to *de novo* Newbler cDNA assemblies for other organisms: N50 = 1,735 bp in *Oncopeltus* *fasciatus* , Ewen-Campen et al. [65]; N50 = 1,333 bp in *Silene* *vulgaris* , Sloan et al. [51]; N50 = 1,588 bp in *Spalax* *galili* , Malik et al. [66]; and N50 = 854 bp in *Arctocephalus* *gazella* , Hoffman & Nichols [67].

We sequenced samples using normalized and non-normalized cDNA pools and examined the influence each protocol had on gene discovery. Following sequencing of the first normalized plate, we used a new protocol from Roche that excluded normalization of libraries. Surprisingly, we found that normalization did not necessarily improve the number of uniquely assembled contigs. Theoretically, normalization reduces the sequencing of overly abundant transcripts and increases the discovery of rare sequences [68,69], but normalization does not disproportionately influence gene discovery when enough sequencing coverage is achieved [70]. We found that read coverage per transcript increased for our non-normalized plates compared to the normalized pilot plate. However, Ekblom et al. [71] suggest that differences in technologies and sequencing effort may ultimately affect comparisons between normalized and non-normalized cDNA libraries, and any differences we identify may be due to different protocols used to extract RNA and prepare pooled libraries.

### Mapping to rodent genomes

The mammalian laboratory models *Mus* and *Rattus* have extensively annotated genomes that provide a good substitute reference for other rodent sequencing projects. The New World *Peromyscus* and Old World *Mus* and *Rattus* lineages last shared a common ancestor ~25 million years ago [72]. Deep divergence and high rates of chromosome evolution across these lineages [73] may have affected the percentage of identified homologous gene transcripts. Ramsdell et al. [74] found the *Peromyscus* genome to be more similar to *Rattus* than *Mus* due to an enhanced level of genome rearrangement in *Mus* compared to ancestral muroids. Our results support these findings given that most *Peromyscus* transcripts mapped to different chromosomes (96.1%) between *Mus* and *Rattus*. Our homologous gene matches between *Peromyscus* and *Rattus* also represented a higher proportion (30.1%) of total *Rattus* genes than homologous gene matches between *Peromyscus* and *Mus* (25.7%). Non-homologous hits and mapping differences between reference genomes may also be due to highly variable or alternatively spliced transcripts, contamination by genomic DNA, or inclusion of low-quality data [75], although our assembly methods included measures to limit the influence of these artifacts.

### Functional annotation and tissue comparisons

Over 75% of our assembled contigs produced significant BLASTX hits to known genes in NCBI’s nonredundant (nr) protein database. This rate of annotation is similar to studies on other non-model species with genomic information available from closely-related model organisms, e.g. 66% in the rodent *Ctenomys* *sociabilis* [76] and 79.7% in the plant *Silene* *vulgaris* [50]. These rates are much higher than some other organisms with few model relatives, such as 19.58% in a bat, *Artibeus* *jamaicensis* [77],, 18% in a butterfly, *Melitaeacinxia* [59],, and 29.2% in the gastropod, *Pomaceacanaliculata* [23]. Phylogenetic analyses support *Peromyscus* spp. and *Cricetulus* spp. as members of a monophyletic clade that diverged separately from *Mus* and *Rattus* [72], and *C* *. griseus* represented the highest proportion of BLASTX top-hits (Figure S2, Supplementary Material). Laboratory use of *C* *. griseus* is not as prevalent as *Mus* or *Rattus*, but Chinese hamster ovary (CHO) cell lines are commonly used *in vitro* to produce biopharmaceuticals [78], and a draft genome has also been sequenced [79]. Research on protein pathways and interactions within CHO cell lines provides a future resource for investigating functional consequences of divergent genes between urban and rural populations of *P* *. leucopus* .

Transcriptome studies in model rodents provide useful context for understanding how much of each tissue-specific transcriptome we sequenced in this study. Yang et al. [80] used microarray analysis to identify 12,845 active genes in *Mus* liver, and RNA-Seq using an Illumina HiSeq 2000 on *Rattus* liver identified 7,514 known genes [81]. Our gene discovery was between 40–60% of these previously reported liver transcriptomes. In brain tissue, 4,508 genes were identified in *Mus* by Yang et al. [80], and Chrast et al. [82] report ~4,000 genes identified in *Mus* brain tissue. The 2,610 gene annotations from our brain cDNA libraries represent between 60–65% of the full *P* *. leucopus* brain transcriptome. Microarray analysis of testis RNA identified up to 13,812 known genes [83] in *Mus* , and 454 sequencing of cDNA libraries from *C* *. griseus* identified 13,187 annotations in ovary [76]. UniGene [84] includes 8,946 genes for *Mus* testis, 5,285 for *Mus* ovaries, 4,355 for *Rattus* testis, and 5,093 for *Rattus* ovaries. The only cDNA library established in UniGene for *Peromyscus* spp. includes 635 putative genes from testis [85]. Our assembled libraries from gonad tissue fall within these ranges, and non-annotated transcripts could represent *Peromyscus* -specific genes. To recover 100% of each tissue transcriptome, samples would need to be prepared at various developmental stages and under various environmental conditions.

Fisher’s Exact Tests allowed us to identify annotated transcripts over-represented in one tissue compared to the others. The brain transcriptome of the social rodent, *C* *. sociabilis* , exhibited highly expressed genes involved with behavior and signal transduction [76]. Over-represented GO terms in *P* *. leucopus* brain tissue were related to similar major functions in the brain, including regulation of behavior, cellular signaling, actin binding, ion transport and channel activity, motor activity, and calcium ion binding. In liver, over-represented GO terms were largely dedicated to metabolic processes including ATP binding, GTP binding, NADH dehydrogenase, and electron carrier activity. There were also several GO terms related to the immune response, hematopoietic processes, and nutrient binding; these annotations are supported by microarray and RNA-seq analyses of liver in mouse and rat, respectively [80,81].

### SNP discovery and characterization

Without a reference genome, aligning reads to assembled transcripts and assigning mismatches as SNPs [86] is an acceptable substitute for generating sequence polymorphisms for non-model species [51,54,87]. Difficulties may persist in distinguishing true SNPs from false positives created by sequencing errors, misaligned reads, or alignment of reads to paralogous genes. Identifying true SNPs depends on assembly quality, filtering criteria of nucleotide mismatches during alignment, and statistical models used to call nucleotide variants [88]. Incorporating a probabilistic framework in SNP-calling algorithms greatly reduces false positives [89,90].

We used conservative filtering criteria when calling SNPs to minimize false positives. SAMtools [91] excels at SNP detection with low sequence coverage by comparing multiple samples simultaneously [89,90]. We also filtered variants based on thresholds of quality and minimum occurrence, and restricted maximum coverage to filter out false positive SNPs from paralogous genes. Excluding transcripts with the highest coverage after mapping limits problems with gene duplications [92]. The thresholds we used for minimum SNP occurrence and nucleotide quality reduce error rates by several orders of magnitude for pooled data, ensuring the reliability of SNP libraries for downstream analyses [93]. Our SNP library represents highly confident variant calls and will serve as an important resource for future population genetic studies of urban and rural populations of *P* *. leucopus* . We cannot completely rule out paralogous genes or misalignments in our transcriptome assemblies, and thus future work will require sequencing of transcripts from multiple individuals to validate SNP calls in candidate genes of particular interest.

### Positive selection and the transcriptome

We used the ratio of non-synonymous to synonymous substitution rates (*p*_N_ ⁄ *p*_S_) to identify candidate genes that may have experienced positive selection in urban populations of *P* *. leucopus* . Using SNPs to calculate (*p*_N_ ⁄ *p*_S_) ratios in ORFs from assembled transcriptomes can be a fruitful method for identifying the operation of natural selection on individual loci [6,52,94]. This approach has recently been used to identify genes under positive or purifying selection between cichlid fish lineages in Nicaragua [56], between lake whitefish species pairs [54], and within an invasive gastropod [23]. Studies traditionally identify positive selection in genes with *p*_N_ ⁄ *p*_S_ > 1.0. We used this cutoff value, but also identified sequence pairs with *p*_N_ ⁄ *p*_S_ between 0.5 and 1.0 to avoid overlooking relevant non-synonymous substitutions in candidate genes that might be of interest for individual re-sequencing projects. Lack of full-length ORFs can decrease *p*_N_ ⁄ *p*_S_ values when some non-synonymous substitutions are unsampled [56,57]. The *p*_N_ ⁄ *p*_S_ index can also be used when samples have been pooled prior to sequencing [95], unlike summary statistics that rely on allele frequencies [51].

We used McDonald-Kreitman tests to further elucidate patterns of evolution in candidate genes. This method can identify adaptive changes between species and primarily detects selection processes occurring thousands or even millions of years in the past. We calculated a neutrality index (NI) as (*p*_N_ ⁄ *p*_S_)/(*d*_N_ ⁄ *d*_S_) to look at deviations from neutral expectations [96,97]. While we detected an excess of non-synonymous polymorphisms within *P* *. leucopus* in genes with functions including demethylation, xenobiotic metabolism, and innate immunity, we did not find evidence of positive selection between species. While these patterns could suggest purifying selection preventing the fixation of harmful mutations [98] or indicate balancing selection acting to maintain favorable alleles in different populations [26], interpretation should proceed cautiously due to limitations of polymorphism data generated from pooled transcriptomes. The inability to assign individual allele frequencies when identifying polymorphisms leads to an ascertainment bias towards high within-species *p*_N_ ⁄ *p*_S_ ratios compared to interspecies ratios, and this bias may explain the lack of NI values < 1 (positive selection). These results could be interpreted as the result of balancing selection whereby different alleles are favored in different urban populations, however, which would seem consistent with the ecology of these relatively isolated populations. Individual resequencing of mice from multiple populations will remove the ascertainment bias, uncover more polymorphisms, and allow the use of more powerful tests to study recent selective pressures in urban populations.

Many ecological changes arising from urbanization may drive local adaption to novel conditions in fragmented urban populations, and we made several predictions about the types of adaptive traits present in urban habitats from current literature. Genes involved in divergence of urban and rural populations of white-footed mice are likely associated with quantitative traits affected by crowded (i.e. high population density) and polluted urban environments (life history, longevity, reproduction, immunity, metabolism, thermoregulatory and / or toxicological traits). We identified candidate genes (*p*_N_ ⁄ *p*_S_ > 1) that supported these predictions between urban and rural populations of mice, but also between individual urban populations. The urban matrix is a strong enough barrier to dispersal that white-footed mouse populations in individual city parks may experience highly localized selective pressures in addition to selective pressures that are general to urban environments [41].

New predators, competitors, parasites, and pathogens can drive local adaptation of traits, especially those related to immunity, in novel urban environments [15,16]. We identified candidate genes involved in the innate immune system and activation of the complement pathway to identify pathogens. Additionally, two candidate genes were identified in comparisons of urban populations that function in blood coagulation and inflammation. The innate immune system is a biochemical pathway that removes pathogens by identifying and killing target cells [99], and positive selection is found to act on pathogen recognition genes within the complement activation pathway [100]. The introduction of invasive species, population growth of ‘urban exploiters’, and increased traffic, trade, and transportation within cities can introduce large numbers of novel pathogens [101], and white-footed mice in NYC may be evolving to efficiently recognize them and respond immunologically. We also identified several genes involved in metabolism that were divergent between populations, and a gene expressed during spermatogenesis that was divergent between urban and rural populations. Rapid evolution has been identified in reproductive proteins between *Peromyscus* spp. affecting spermatogenesis, sperm competition, and sperm-egg interactions [102], and the intensity of sperm competition and reproductive conflict may be increasing in dense *P* *. leucopus* populations in NYC.

Increasing air, water, and soil pollution are all typical impacts of urbanization [17–19]. One potential marker of increased exposure to pollutants is hypermethylation of regulatory regions of the genome [17,103–105]. Positive selection may also be acting on genes involved in xenobiotic metabolism. Heavy metals including mercury, lead, and arsenic occur at increased concentrations within NYC park soils (S. Harris, unpublished data), and McGuire et al. [106] found lower pH and higher concentrations of heavy metals in NYC parks compared to green roofs. PCB resistance was identified in multiple populations of *Fundulus* *heteroclitus* [18], and Wirgin et al. [107] also found rapid PCB resistance in tomcod from the Hudson River through positive selection. In urban to rural comparisons we found two potential toxicological candidate genes: one gene involved in metabolizing foreign chemical compounds (i.e. xenobiotics), and a demethylase that removes methyl groups from histone lysines.

Comparing candidate genes from all pairwise analyses with *p*_N_ ⁄ *p*_S_ between 0.5 and 1 reveals several additional patterns. Proteins were identified that function in the alternative pathway, which acts continuously in an organism without antibody activation to clear foreign pathogens [108], and supports our conclusion that positive selection (*p*_N_ ⁄ *p*_S_ > 1) is acting on the innate immune system in these populations. Four *cytochrome p450* genes, *2d27-like*, family *2 subfamily B*, *subfamily polypeptide 13*, and *2a15*, exhibited *p*_N_ ⁄ *p*_S_ between 0.5 and 1 in urban populations and between urban and rural populations. *Cytochrome p450 2a15* was also found to have a *p*_N_ ⁄ *p*_S_ > 1 and McDonald-Kreitman tests found significantly more polymorphisms within *P* *. leucopus* than between species (Table 4, Table 5). The *cytochrome p450* family of genes plays a major role in xenobiotic metabolism, including detoxification in variable environments [109,110]. Patterns of divergence and positive selection have been robustly identified in *cytochrome p450* genes in natural populations of both *Mus musculus* when ingesting toxins through their diet and *Tetrahymena thermophila* exposed to toxic environments [110,111]. *P* *. leucopus* in NYC populations may be experiencing different dietary demands and exposure to pollutants, leading to selective pressures on detoxifying genes like the cytochrome p450 gene family.

Alternatively, genetic differences between urban and rural populations may result from genetic drift rather than selection. We will differentiate between drift and selection in future work by examining genetic divergence between multiple urban and rural populations at these candidate loci and additional genes. We must also be cautious when inferring the function of candidate genes after identifying statistical signatures of positive selection.

While a *p*_N_ ⁄ *p*_S_ ratio > 1.0 can represent positive selection, it may also occur due to relaxation of purifying selection, and individual codons within a gene can have an excess of non-synonymous substitutions due to random biological processes [112]. However, current statistical tests address these issues and are generally robust in identifying positive selection [113]. In the case of a single population, *p*_N_ ⁄ *p*_S_ > 1 may not represent positive selection. Kryazhimskiy & Plotkin [114] demonstrated that the relationship between *p*_N_ ⁄ *p*_S_ and selection is radically different when samples originated from the same population; *p*_N_ ⁄ *p*_S_ actually decreases in response to positive selection. To infer selection between two samples using *p*_N_ ⁄ *p*_S_, samples must come from reproductively isolated populations with fixed substitutions [114]. All samples used to calculate *p*_N_ ⁄ *p*_S_ for this study came from reproductively isolated and genetically structured populations [40]. We assembled transcriptome datasets individually for each population to identify fixed substitutions between populations and avoid randomly segregating SNPs in *p*_N_ ⁄ *p*_S_ analyses. Indices such as *p*_N_ ⁄ *p*_S_ identify genes with previously unknown signatures of selection, but candidates still need to be studied in a controlled setting to identify phenotype and function [113].

The ability of *p*_N_ ⁄ *p*_S_ and McDonald-Kreitman tests to detect genes under positive selection is limited in some situations, so it is likely that we have missed many candidate genes. Additionally, such analyses do not identify adaptive variation in gene regulatory regions as opposed to transcribed cDNA [115]. Ratios such as *p*_N_ ⁄ *p*_S_ may also vary widely when there are relatively few mutations per gene [56,108]. Given strong selection within populations, however, it is plausible that multiple substitutions may rise to high frequency or become fixed within a few hundred generations (i.e. in the timeframe of divergence for urban and rural populations of white-footed mice). The candidate genes identified herein can be confirmed in future work using the reference genome of *P* *. maniculatus* (sequenced and currently being assembled) and multiple tests of selection that provide more statistical power and higher resolution when identifying types and age of selection in single candidate genes [116,117]. These emerging resources will allow us to validate many of our predicted polymorphisms, identify paralogous genes with greater certainty, and perform more powerful tests of selection by providing genetic distances and genomic coordinates for our sequenced contigs. Our ongoing work in this system uses these external resources with our new transcriptomic and genomic libraries from individual mice from several urban, rural, and suburban populations. These ongoing studies employ multiple outlier statistics based on the allele frequency spectrum and linkage disequilibrium to examine recent selection in both coding and non-coding regions of urban white-footed mouse genomes.

## Materials and Methods

### Ethics statement

All animal procedures were approved by the Institutional Animal Care and Use Committee at Brooklyn College, CUNY (Protocol No. 247), and adhered to the Guidelines of the American Society of Mammalogists for the Use of Wild Mammals in Research [118]. Field work was conducted with the permission of the New York State Department of Environmental Conservation (License to Collect or Possess Wildlife No. 1603) and the New York City Department of Parks and Recreation.

### Study Sites and population sampling

**Figure 6:**
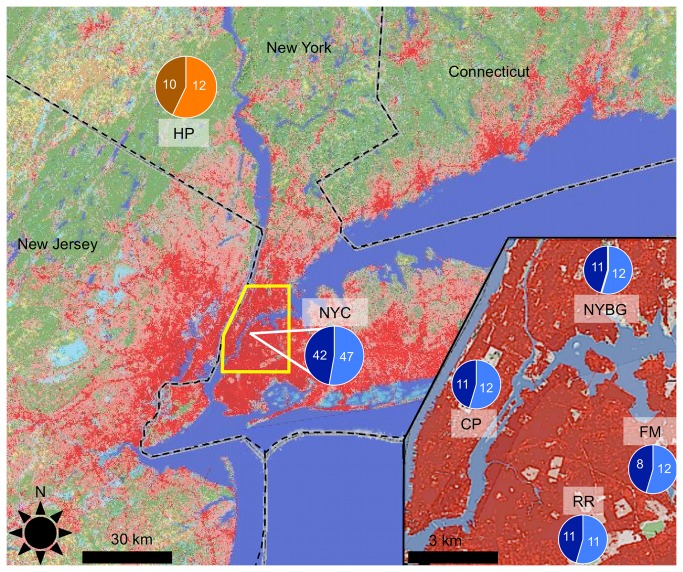
Location and number of individuals collected from five populations in the NYC metropolitan area. Urban populations are in shades of blue; light blue = male; dark blue = female. Rural population in orange and brown; orange = male; brown = female. Areas shaded red on the map indicate degree of urbanization (i.e. impermeable surface cover such as roads and rooftops) and green areas indicate vegetation cover from the 2006 National Landcover Database (CP = Central Park; NYBG = New York Botanical Gardens; RR = Ridgewood Reservoir; FM = Flushing Meadows-Willow Lake; HP = Harriman State Park).

*P* *. leucopus* were trapped and collected from each of four urban and one rural site (*N* = 20-25 / population) for sequencing and analysis (total *N* = 112; Figure 6). The four urban sites (Central Park, Flushing Meadows-Willow Lake, New York Botanical Gardens, and the Ridgewood Reservoir) were chosen due to their large area, isolation by dense urban matrix, high population density of mice, substantial genetic differentiation, and genetic isolation from other populations [40,41]. The rural site, Harriman State Park located ~68 km north of Central Park, is one of the largest contiguous protected areas nearby and the most likely representative of a non-urban population of mice in proximity to NYC. Mice were trapped over a period of 1-3 nights at each site using four 7x7 transects of 3” x3” x9” Sherman live traps. Mice were killed by cervical dislocation and immediately dissected in the field. Livers, gonads and brains were extracted, rinsed with PBS to remove any debris from the surface of the tissue, and immediately placed in RNALater^®^ (Ambion Inc., Austin, TX) on ice before transport and storage at -80°C. These tissue types were chosen for initial analysis due to their wide range of expressed gene transcripts [78] and potential roles in adaptation to urban conditions.

### RNA extraction and cDNA library preparation

Total RNA was extracted and cDNA libraries were pooled for all five populations for four multiplexed plates of 454 sequencing. The first plate of sequencing was normalized to produce equalized concentrations of all transcripts present, potentially allowing enhanced gene discovery and greater overall coverage of the transcriptome [119]. However, the normalization process introduces additional steps and biases in library preparation [50], and resulted in a relatively low number of total high-quality 454 reads. Thus, non-normalized libraries were prepared using a modified protocol for the last three 454 plates.

For plate 1, total RNA was isolated from ~60 mg of liver (eight males and eight females / population), ~60 mg of testis (eight males / population), and ~60 mg of ovaries (eight females / population) for two populations using RNaqueous^®^ kits (Ambion, Austin, TX). Individual RNA extracts were pooled by population and organ type and selected for mature mRNA using the MicroPoly(A) Purist^™^ kit (Ambion, Austin, TX). Next, mRNA pools were reverse-transcribed using the SMARTer^™^ cDNA synthesis kit (Clontech, Mountain View, CA), and normalized using the Trimmer-Direct cDNA normalization kit (Evrogen, Moscow, Russia). Then, normalized cDNA pools were sequenced with multiplex identifiers using standard 454 FLX Titanium protocols. This pilot plate contained cDNA pools for Harriman State Park and Flushing Meadows-Willow Lake.

For plates 2-4, total RNA using Trizol^®^ reagent (Invitrogen, Carlsbad, CA) was extracted from ~70 mg of brain tissue (four males and four females / population), ~70 mg of testes (eight males / population), and ~15 mg of liver (four males and four females / population). After DNAse treatment (Promega, Madison, WI) and pooling individual samples in equimolar amounts by population and tissue, the samples were treated with the RiboMinus^™^ Eukaryote kit (Invitrogen, Carlsbad, CA) to reduce ribosomal RNA. RNA pools were then reverse-transcribed using the Roche cDNA synthesis kit (Roche Diagnostics, Indianapolis, IN) and sequenced with multiplex identifiers using standard 454 FLX Titanium protocols. Plate 2 included brain cDNA pools for all five populations, plate 3 included liver and testis cDNA for Central Park, Ridgewood Reservoir, New York Botanical Gardens, and Harriman State Park, and plate 4 included liver cDNA pools from all five populations. All raw sequencing files have been deposited in the GenBank Sequence Read Archive (SRA) under accession number SRP020005.

### Transcriptome assembly

Two methods were used to assemble the best transcriptome from all four 454 plates: Cap3 [120] a long-read assembler that performs well in transcriptome assemblies [60], and Roche’s proprietary software, Newbler (Version 2.5.3), that was designed specifically for assembling 454 sequencing reads with additional features for cDNA sequence. Newbler’s cDNA options assemble reads into contigs, followed by assembly into larger ‘isotigs’ representing alternatively-spliced transcripts. Isotigs are then clustered into larger ‘isogroups’ representing full-length genes. Transcriptome assembly was attempted with the full set of reads using Cap3 and Newbler with cDNA options, but due to computational limitations the full dataset could not be assembled with either software program. We addressed this issue by first assembling sequences from all four plates with Newbler using the genome assembly settings and default parameters after trimming 454 adaptors and bar codes from the reads. Reads that were either ‘assembled’ or ‘partially assembled’ in this pilot run were filtered and used as input for cDNA assemblies in Newbler or Cap3. These reads were filtered from the raw sff files using a locally-installed instance of Galaxy [121]. Before the cDNA assembly, nucleotides with poor quality scores, primer sequences, and long poly(A) tails were removed using cutadapt (Version 1.2.1 2012 [122], and the trim-fastq.pl perl script implemented in Popoolation [93]. The filtered fastq files were then used as input for Cap3 or Newbler with the cDNA assembly option, using default parameters for both assemblies. These assemblies (1. genome assembly with Newbler, 2. cDNA assembly with Newbler, and 3. cDNA assembly with Cap3) were compared to identify the best full reference transcriptome for downstream analysis.

For analyses of individual tissues, separate cDNA assemblies were performed. Tissues were bar-coded, and sequence reads originating from liver, gonads, or brains were parsed from the raw 454 sequencing reads. These datasets were small enough to be assembled separately as tissue-specific transcriptomes in Newbler using the cDNA option with default parameters. Population-specific transcriptomes were also assembled, using the same methodology, to examine population-specific statistical signatures of selection.

### Alignment to model rodent genomes

*Peromyscus* assemblies were initially characterized and annotated by performing two separate analyses using *Mus musculus* and *Rattus norvegicus* genomic resources. The first analysis was used to determine the number of likely genes in each assembly. BLASTN searches were performed against *Mus musculus* (NCBI Annotation Release 103) and *Rattus norvegicus* (NCBI build 5.1) cDNA reference libraries downloaded from NCBI. BLASTN matches were considered significant when sequence identity was greater than 80%, alignment length was at least 50% of the total length of either the query or subject sequence, and the *e*-value was less than 10^-5^. While significant, these hits may not be ideal for population genomic analyses due to inclusion of paralogous gene matches, matches between multi-gene families, and false positive orthologous gene matches. In order to identify individual isotigs representing a single gene with known function useful for statistical analysis, BLASTN results were further filtered by including query hits that matched only one subject ID (i.e. gene) and *vice versa*. These contigs were annotated as ‘Gene Candidates’.

The distribution of *P* *. leucopus* isotigs across model rodent genomes was analzyed. All *P* *. leucopus* isotigs were mapped to chromosomes in the *Mus* (GRCm38) and *Rattus* (RGSC 5.0) reference genomes. Default BLAT parameters were used with an exception for aligning mRNA to genomes across species (-q=rnax -t = dnax [123]), and best BLAT hits were parsed based on percent identity and score (# match − # mismatch).

### Mapping and SNP discovery

To generate a SNP library for downstream population genomic analysis, 454 reads were first mapped to the Newbler cDNA assembly using the BWA-SW (http://bio-bwa.sourceforge.net/) alignment algorithm for long read mapping [124]. We only used trimmed reads from the final assembly, removed singletons before mapping to reduce false positive SNP calls from sequencing errors or duplicate reads, and included reads with a mapping quality > 20 in SAMtools. The SAM file from BWA-SW was used in the SAMtools package (v. 0.1.17 [89]) to call SNPs using the mpileup command with a maximum coverage cutoff of 200. The SNP calling pipeline implemented in SAMtools uses base alignment quality (BAQ) calculations to generate likelihoods of genotypes, can overcome low coverage by using sequence information from multiple samples to call variants, and uses Bayesian inference to make SNP calls with high confidence [87–89]. In addition to the default parameters in SAMtools, we included stringent additional filters by removing any potential INDELs, only including SNPs with a phred quality (Q-value) ≥ 20, a minimum occurrence of two, and coverage ≤ 200 to exclude alignment artifacts, duplicates, and paralogous genes [93,118,124–126].

### Functional annotation of transcriptomes

The reference transcriptome was annotated by performing a BLASTX search to identify homologous sequences from the NCBI non-redundant protein database, and then GO terms associated with BLASTX hits were retrieved using the annotation pipeline in Blast2GO [125,126]. Tissue-specific assemblies were also annotated in Blast2GO, and Fisher’s Exact Test was used to examine whether GO terms were over-represented between pairs of tissue types. Each pairwise tissue comparison (liver, brain, gonad) was analyzed for over-representation, and significant results were identified with a False Discovery Rate (FDR) ≤ 0.05.

### Prediction of Open Reading Frames (ORFs) and p_N_ ⁄ p_S_ calculations

Regions containing ORFs were identified using BLASTX searches of our assembled contigs against the NCBI non-redundant protein database. Only best hits with an *e*-value ≤ 10^-5^, and when query transcripts hit only one subject sequence and *vice versa*, were kept. From these results, a general feature file (GFF) was manually created indicating the start and stop coordinates, strand information, and reading frame from the BLASTX results. Within these protein coding regions, putative ORFs were identified when a start codon was found and the reading frame was greater than 150 bp long. The Perl script, Syn-nonsyn-at-position.pl, implemented in Popoolation v. 1.2.2 [93] was used to define population-specific SNPs obtained from the SAMtools analysis above as either non-synonymous or synonymous.

The ratio of non-synonymous (*p*_N_) to synonymous (*p*_S_) SNP substitutions (*p*_N_ ⁄ *p*_S_) was calculated between individual Newbler cDNA population assemblies to identify coding sequences potentially experiencing directional selection in urban *P* *. leucopus* populations. For each population pair, the fastaFromBed command in bedtools [127] was used to filter contigs and generate a fasta file of putative ORFs (identified above) for each population assembly. The USEARCH (http://www.drive5.com/usearch/) clustering and alignment software for genomic datasets [128] was used to create pairwise alignments between all population ORFs using an *e*-value ≤ 0.001. Signatures of selection between aligned ORFs were identified using KaKs_Calculator1.2 (Model GY) [129] to calculate the ratio of non-synonymous (*p*_N_) to synonymous (*p*_S_) SNPs in each population pair. Only transcripts with at least three SNPs, an ORF length greater than 150 bp, and no in-frame stop codons were included. The mean number of SNPs per ORF was 1.4 ± SE = 2.9. A three SNP threshold was chosen to avoid bias as Ka / Ks calculations lose statistical power as the number of substitutions per ORF decreases [130]. The maximum likelihood method was used that accounts for evolutionary characteristics (i.e. ratio of transition / transversion rates, nucleotide frequencies) of our transcriptome datasets. Contigs with elevated *p*_N_ ⁄ *p*_S_ ratios were then annotated in Blast2GO as above.

Candidate genes were screened for evidence of recombination, and additional signatures of natural selection were examined using McDonald-Kreitman tests. We used BLASTN searches to find orthologous mRNA sequences from multiple species for each candidate gene. For recombination analysis, multiple mammals were used and always included *Cricetulus* *griseus* , *Rattus* *norevegicus* , or *Mus musculus*. Orthologous sequences were codon-aligned using MACSE [131] and then scanned for evidence of recombination using a GARD analysis implemented in the Data Monkey webserver [132,133]. For McDonald-Kreitman tests, orthologous genes between *Peromyscus* *leucopus* and *Rattus norvegicus* or *Cricetulus* *griseus* were codon aligned with MACSE [131]. Non-overlapping datasets of polymorphisms within *P* *. leucopus* and fixed genetic changes between species were then generated. The McDonald-Kreitman test was performed with these data using DnaSP v. 5.10.1 [134]. Fasta files of assembled contigs / isotigs, vcf files of SNP marker data, BLAST2GO files of functional annotations, and output files from population genetics tests are available on the Dryad digital repository (doi: 10.5061/dryad. r8ns3).

## Supporting Information

Figure S1 **Frequency distribution of depth of coverage (reads / contig).** (a) The Newbler cDNA assembly. Red line indicates median coverage = 4.9 reads, Interquartile range (IQR) = 4.1. (b) The Newbler genomic assembly, median = 4.7 reads, IQR = 4.6. (c) The Cap3 assembly, median = 5.0 reads, IQR = 7.0.
(TIF)

Figure S2 **Distribution of species with the most top-hit BLASTX results in Blast2Go using the Newbler cDNA assembly as the query.**
(TIF)

Table S1 **Sequencing and assembly statistics for Newbler cDNA transcriptome assembly by tissue type and 454 sequencing plate.**
(DOCX)

Table S2 **Full list of over represented GO terms for all tissue pairwise comparisons from Fisher’s Exact Test (FDR ≤ 0.5). (a) Liver. (b) Brain. (c) Gonads.**
(DOCX)

Table S3 **Candidate loci with *p*_N_ ⁄ *p*_S_ between 0.5 and 1.**
(DOCX)
