# Supplementary figures and images for: Signatures of Rapid Evolution in Urban and Rural Transcriptomes of White-Footed Mice (Peromyscus leucopus) in the New York Metropolitan Area
Source: PLoS One. 2013 Aug 28;8(8):e74938. doi: 10.1371/journal.pone.0074938 (PMC3756007; doi:10.1371/journal.pone.0074938)

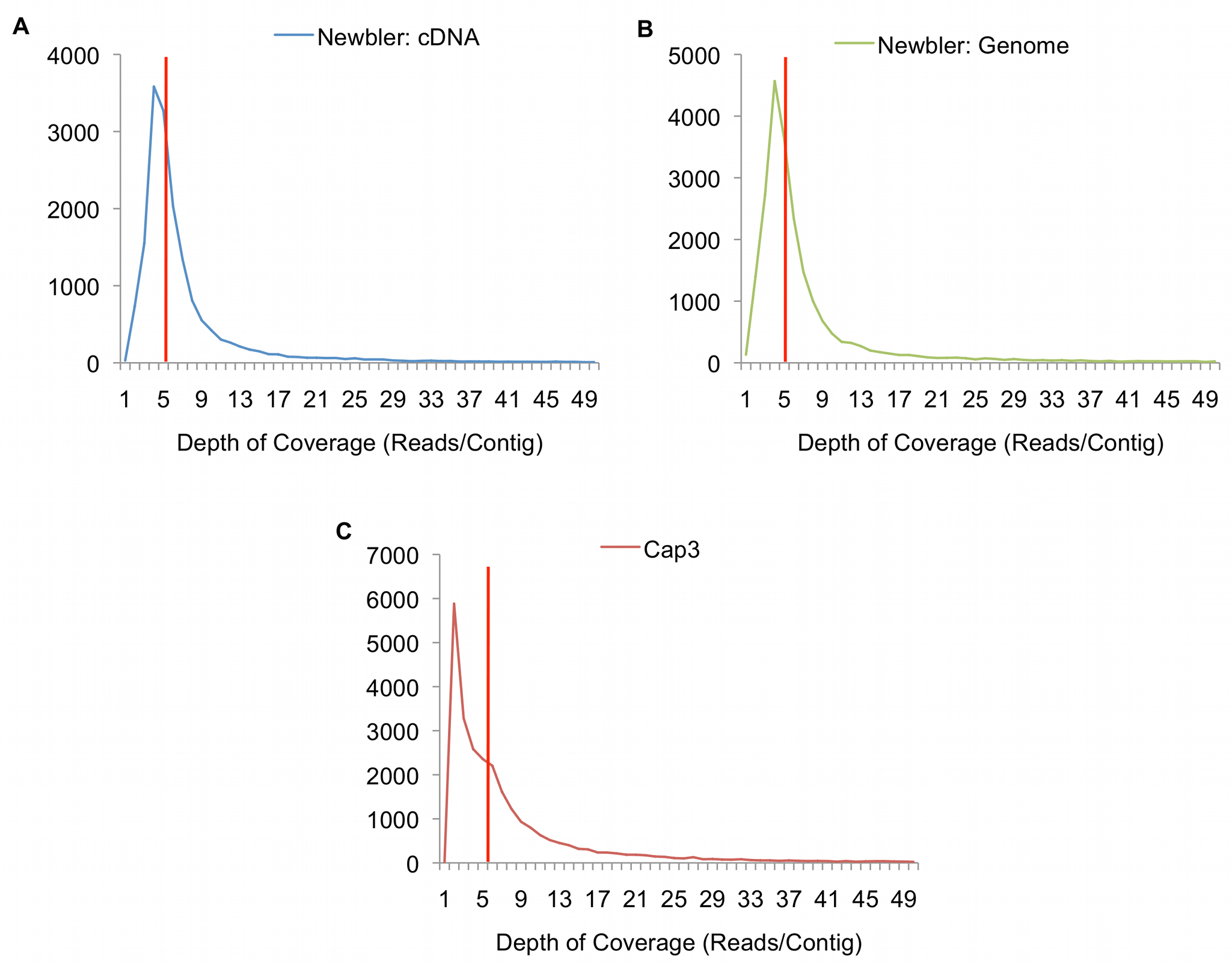

Supplement: Figure S1 — Frequency distribution of depth of coverage (reads / contig). (a) The Newbler cDNA assembly. Red line indicates median coverage = 4.9 reads, Interquartile range (IQR) = 4.1. (b) The Newbler genomic assembly, median = 4.7 reads, IQR = 4.6. (c) The Cap3 assembly, median = 5.0 reads, IQR = 7.0. (TIF) [file pone.0074938.s001.tif]

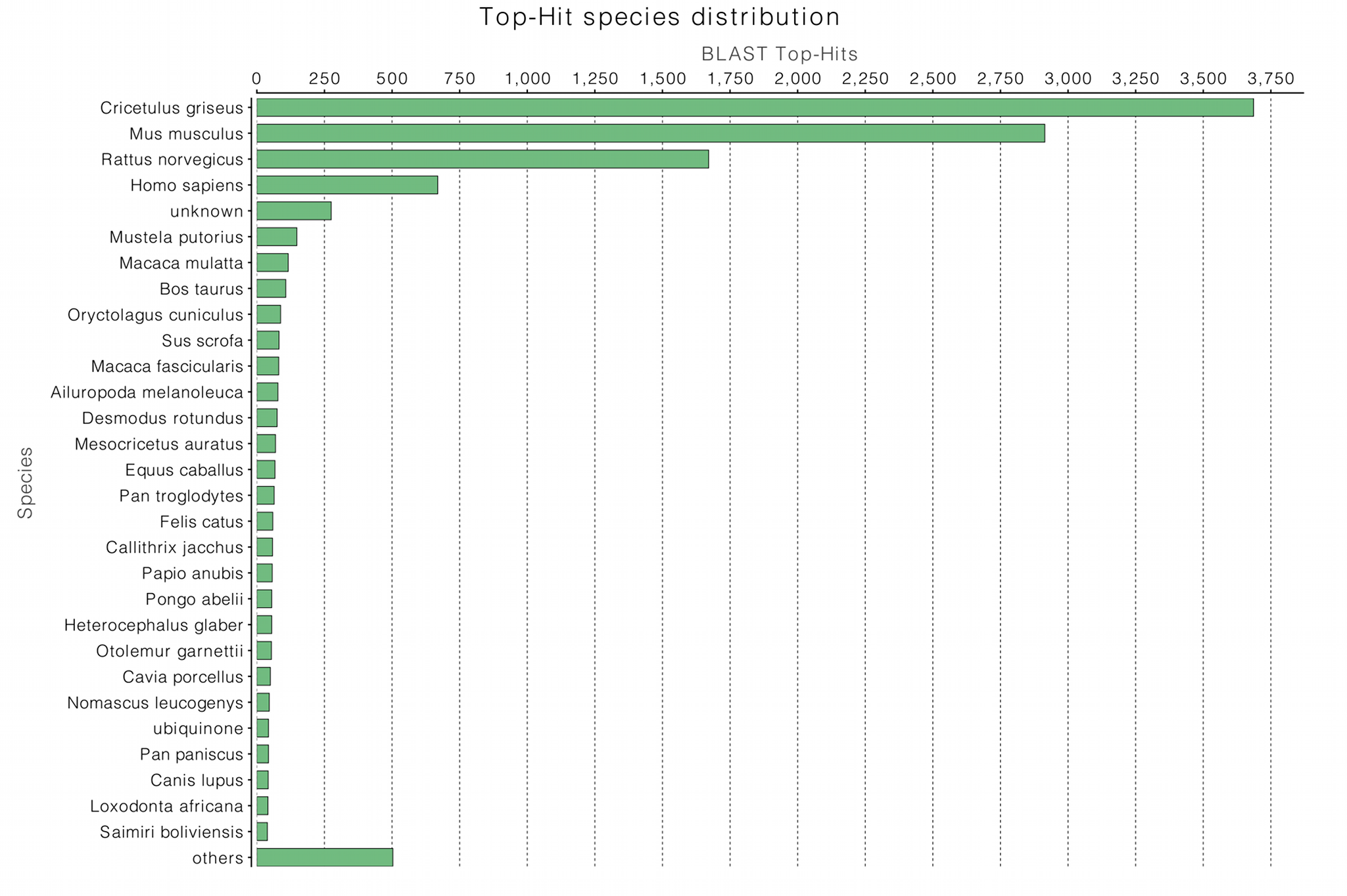

Supplement: Figure S2 — Distribution of species with the most top-hit BLASTX results in Blast2Go using the Newbler cDNA assembly as the query. (TIF) [file pone.0074938.s002.tif]
