# Supplementary material for: Signatures of Rapid Evolution in Urban and Rural Transcriptomes of White-Footed Mice (Peromyscus leucopus) in the New York Metropolitan Area
Source: PLoS One. 2013 Aug 28;8(8):e74938. doi: 10.1371/journal.pone.0074938 (PMC3756007; doi:10.1371/journal.pone.0074938)
Supplement: Table S1 — Sequencing and assembly statistics for Newbler cDNA transcriptome assembly by tissue type and 454 sequencing plate. (DOCX) [file pone.0074938.s003.docx]

|  | Liver | | | Brain | Ovaries | Testis | |
| --- | --- | --- | --- | --- | --- | --- | --- |
|  | Plate 1 | Plate 3 | Plate 4 | Plate 2 | Plate 1 | Plate 1 | Plate 3 |
| Total # reads | 191,244 | 775,896 | 701,974 | 722,086 | 119,700 | 97,568 | 444,172 |
| # Reads used in assembly | 86,232 | 68,329 | 48,677 | 60,406 | 35,295 | 39,507 | 59,425 |
| # Contigs containing reads | 8,177 | 8,564 | 6,260 | 9,858 | 7,085 | 7,260 | 10,764 |
