# Supplementary material for: Signatures of Rapid Evolution in Urban and Rural Transcriptomes of White-Footed Mice (Peromyscus leucopus) in the New York Metropolitan Area
Source: PLoS One. 2013 Aug 28;8(8):e74938. doi: 10.1371/journal.pone.0074938 (PMC3756007; doi:10.1371/journal.pone.0074938)
Supplement: Table S2 — Full list of over represented GO terms for all tissue pairwise comparisons from Fisher’s Exact Test (FDR ≤ 0.5). (a) Liver. (b) Brain. (c) Gonads. (DOCX) [file pone.0074938.s004.docx]

| Liver to Brain | | | Liver to Gonads | | |
| --- | --- | --- | --- | --- | --- |
| GO Term | FDR | # Sequences | GO Term | FDR | # Sequences |
| ATP binding | 5.31E-24 | 184 | ATP binding | 1.75E-62 | 184 |
| zinc ion binding | 5.93E-20 | 154 | zinc ion binding | 4.37E-52 | 154 |
| transcription factor complex | 3.91E-19 | 148 | transcription factor complex | 5.31E-50 | 148 |
| electron carrier activity | 8.53E-18 | 251 | structural constituent of ribosome | 2.39E-39 | 117 |
| structural constituent of ribosome | 5.51E-15 | 117 | soluble fraction | 1.60E-32 | 97 |
| integral to plasma membrane  **A** | 1.40E-14 | 114 | microsome | 8.86E-28 | 83 |
| soluble fraction | 2.35E-12 | 97 | protein homodimerization activity | 4.16E-27 | 81 |
| response to drug | 2.59E-11 | 89 | perinuclear region of cytoplasm | 4.73E-23 | 69 |
| electron transport | 8.49E-11 | 85 | GTP binding | 1.05E-20 | 62 |
| translational elongation | 1.13E-10 | 84 | electron carrier activity | 6.56E-20 | 251 |
| viral transcription | 1.13E-10 | 84 | oxygen binding | 6.72E-20 | 93 |
| microsome | 1.53E-10 | 83 | ubiquitin-protein ligase activity | 5.56E-14 | 42 |
| protein homodimerization activity | 2.75E-10 | 81 | GTPase activity | 5.56E-14 | 42 |
| Golgi membrane | 3.71E-10 | 80 | NADH dehydrogenase (ubiquinone) activity | 2.58E-13 | 40 |
| oxygen binding | 1.97E-09 | 93 | sequence-specific DNA binding | 5.52E-13 | 39 |
| endocrine pancreas development | 3.01E-09 | 73 | drug binding | 5.52E-13 | 39 |
| transcription factor binding | 4.04E-09 | 72 | double-stranded DNA binding | 1.19E-12 | 38 |
| translational termination | 4.04E-09 | 72 | mitochondrial respiratory chain complex I | 2.55E-12 | 37 |
| perinuclear region of cytoplasm | 9.92E-09 | 69 | transcription coactivator activity | 2.55E-12 | 37 |
| protein complex binding | 3.24E-08 | 65 | catalytic step 2 spliceosome | 5.49E-12 | 36 |
| response to metal ion | 3.24E-08 | 65 | heme binding | 1.18E-11 | 35 |
| nuclear mRNA splicing, via spliceosome | 5.71E-08 | 63 | microtubule | 1.18E-11 | 35 |
| negative regulation of cell proliferation | 5.71E-08 | 63 | apical plasma membrane | 1.18E-11 | 35 |
| cellular membrane organization | 5.71E-08 | 63 | nuclear speck | 1.18E-11 | 35 |
| aging | 7.64E-08 | 62 | unfolded protein binding | 1.17E-10 | 32 |
| GTP binding | 7.64E-08 | 62 | cytosolic small ribosomal subunit | 1.17E-10 | 32 |
| sodium ion transport | 1.86E-07 | 59 | protein heterodimerization activity | 1.17E-10 | 32 |
| anti-apoptosis | 2.50E-07 | 58 | early endosome | 2.51E-10 | 31 |
| protein domain specific binding | 3.37E-07 | 57 | protein C-terminus binding | 2.47E-09 | 28 |
| positive regulation of cell proliferation | 4.54E-07 | 56 | cytosolic large ribosomal subunit | 2.47E-09 | 28 |
| negative regulation of signal transduction | 1.49E-06 | 52 | basement membrane | 2.40E-08 | 25 |
| chromatin | 2.01E-06 | 51 | serine-type endopeptidase activity | 2.40E-08 | 25 |
| endosome membrane | 3.62E-06 | 49 | external side of plasma membrane | 5.11E-08 | 24 |
| positive regulation of transcription from RNA polymerase II promoter | 8.79E-06 | 46 | mRNA binding | 5.11E-08 | 24 |
| DNA repair | 8.79E-06 | 46 | translation initiation factor activity | 5.11E-08 | 24 |
| response to organic cyclic compound | 8.79E-06 | 46 | chaperone binding | 1.09E-07 | 23 |
| in utero embryonic development | 8.79E-06 | 46 | heparin binding | 1.09E-07 | 23 |
| cell division | 1.58E-05 | 44 | magnesium ion binding | 1.09E-07 | 23 |
| response to glucocorticoid stimulus | 2.12E-05 | 43 | tubulin complex | 2.31E-07 | 22 |
| ubiquinone biosynthetic process | 2.12E-05 | 43 | endoplasmic reticulum lumen | 4.93E-07 | 21 |
| ubiquitin-protein ligase activity | 2.82E-05 | 42 | centrosome | 4.93E-07 | 21 |
| GTPase activity | 2.82E-05 | 42 | flavin adenine dinucleotide binding | 1.04E-06 | 20 |
| mitosis | 2.82E-05 | 42 | nuclear membrane | 1.04E-06 | 20 |
| fatty acid biosynthetic process | 2.82E-05 | 42 | lysosomal membrane | 1.04E-06 | 20 |
| G-protein coupled receptor signaling pathway | 2.82E-05 | 42 | phosphatidylinositol binding | 2.21E-06 | 19 |
| regulation of cellular localization | 3.74E-05 | 41 | cytochrome-c oxidase activity | 2.21E-06 | 19 |
| sensory perception | 3.74E-05 | 41 | transcription corepressor activity | 2.21E-06 | 19 |
| mitochondrial electron transport, NADH to ubiquinone | 3.74E-05 | 41 | coated pit | 2.21E-06 | 19 |
| ATP catabolic process | 3.74E-05 | 41 | protease binding | 2.21E-06 | 19 |
| actin cytoskeleton | 3.74E-05 | 41 | respiratory chain complex IV | 2.21E-06 | 19 |
| response to hypoxia | 3.74E-05 | 41 | ubiquitin protein ligase binding | 4.69E-06 | 18 |
| xenobiotic metabolic process | 3.74E-05 | 41 | mitochondrial large ribosomal subunit | 4.69E-06 | 18 |
| positive regulation of cellular component organization | 5.01E-05 | 40 | melanosome | 4.69E-06 | 18 |
| post-translational protein modification | 5.01E-05 | 40 | microtubule binding | 9.89E-06 | 17 |
| NADH dehydrogenase (ubiquinone) activity | 5.01E-05 | 40 | calmodulin binding | 9.89E-06 | 17 |
| neuronal cell body | 5.01E-05 | 40 | pyridoxal phosphate binding | 9.89E-06 | 17 |
| sequence-specific DNA binding | 6.65E-05 | 39 | rRNA binding | 9.89E-06 | 17 |
| isomerase activity | 6.65E-05 | 39 | small nuclear ribonucleoprotein complex | 9.89E-06 | 17 |
| drug binding | 6.65E-05 | 39 | hydro-lyase activity | 2.08E-05 | 16 |
| peptide binding | 6.65E-05 | 39 | aromatase activity | 2.08E-05 | 16 |
| DNA replication | 6.65E-05 | 39 | immunoglobulin complex | 2.08E-05 | 16 |
| S phase of mitotic cell cycle | 6.65E-05 | 39 | trans-Golgi network | 2.08E-05 | 16 |
| secondary metabolic process | 7.00E-05 | 167 | metalloendopeptidase activity | 2.08E-05 | 16 |
| response to toxin | 8.90E-05 | 38 | heat shock protein binding | 2.08E-05 | 16 |
| double-stranded DNA binding | 8.90E-05 | 38 | oxidoreductase activity, acting on paired donors, with incorporation or reduction of molecular oxygen, NADH or NADPH as one donor, and incorporation of one atom of oxygen | 4.33E-05 | 15 |
| mitochondrial respiratory chain complex I | 1.18E-04 | 37 | copper ion binding | 4.33E-05 | 15 |
| late endosome | 1.18E-04 | 37 | alcohol sulfotransferase activity | 4.33E-05 | 15 |
| transcription coactivator activity | 1.18E-04 | 37 | ribosome binding | 4.33E-05 | 15 |
| cellular amino acid biosynthetic process | 1.18E-04 | 37 | platelet alpha granule lumen | 4.33E-05 | 15 |
| spermatogenesis | 1.18E-04 | 37 | S-adenosylmethionine-dependent methyltransferase activity | 4.33E-05 | 15 |
| axon guidance | 1.58E-04 | 36 | ruffle | 4.33E-05 | 15 |
| catalytic step 2 spliceosome | 1.58E-04 | 36 | mitochondrial nucleoid | 4.33E-05 | 15 |
| platelet activation | 1.58E-04 | 36 | monosaccharide binding | 4.33E-05 | 15 |
| protein kinase binding | 1.58E-04 | 36 | sodium ion transmembrane transporter activity | 8.98E-05 | 14 |
| dendrite | 1.58E-04 | 36 | fatty acid binding | 8.98E-05 | 14 |
| anaphase-promoting complex-dependent proteasomal ubiquitin-dependent protein catabolic process | 1.58E-04 | 36 | collagen | 8.98E-05 | 14 |
| steroid biosynthetic process | 1.58E-04 | 36 | nucleosome | 8.98E-05 | 14 |
| positive regulation of cell differentiation | 1.58E-04 | 36 | integral to endoplasmic reticulum membrane | 8.98E-05 | 14 |
| heme binding | 2.10E-04 | 35 | late endosome membrane | 8.98E-05 | 14 |
| transmembrane signaling receptor activity | 2.10E-04 | 35 | growth factor binding | 8.98E-05 | 14 |
| negative regulation of ubiquitin-protein ligase activity involved in mitotic cell cycle | 2.10E-04 | 35 | transcriptional repressor complex | 8.98E-05 | 14 |
| microtubule | 2.10E-04 | 35 | bile-salt sulfotransferase activity | 8.98E-05 | 14 |
| negative regulation of endopeptidase activity | 2.10E-04 | 35 | thiolester hydrolase activity | 1.84E-04 | 13 |
| apical plasma membrane | 2.10E-04 | 35 | 3'-phosphoadenosine 5'-phosphosulfate binding | 1.84E-04 | 13 |
| integral to organelle membrane | 2.10E-04 | 35 | aminoacyl-tRNA ligase activity | 1.84E-04 | 13 |
| nuclear speck | 2.10E-04 | 35 | NAD binding | 1.84E-04 | 13 |
| negative regulation of transport | 2.78E-04 | 34 | N-acetyltransferase activity | 1.84E-04 | 13 |
| protein homooligomerization | 2.78E-04 | 34 | very-low-density lipoprotein particle | 1.84E-04 | 13 |
| positive regulation of ubiquitin-protein ligase activity involved in mitotic cell cycle | 2.78E-04 | 34 | lamellipodium | 1.84E-04 | 13 |
| lipoprotein metabolic process | 2.78E-04 | 34 | mitochondrial small ribosomal subunit | 1.84E-04 | 13 |
| ribonucleoprotein complex assembly | 2.78E-04 | 34 | carboxylesterase activity | 1.84E-04 | 13 |
| positive regulation of cell migration | 2.78E-04 | 34 | microtubule motor activity | 1.84E-04 | 13 |
| rRNA transcription | 2.78E-04 | 34 | transcription activator activity | 1.84E-04 | 13 |
| acyl-carrier-protein biosynthetic process | 4.98E-04 | 32 | Ras GTPase activator activity | 1.84E-04 | 13 |
| unfolded protein binding | 4.98E-04 | 32 | U12-type spliceosomal complex | 3.87E-04 | 12 |
| proteasome complex | 4.98E-04 | 32 | protein kinase regulator activity | 3.87E-04 | 12 |
| cytosolic small ribosomal subunit | 4.98E-04 | 32 | single-stranded DNA binding | 3.87E-04 | 12 |
| mitochondrial outer membrane | 4.98E-04 | 32 | peptidyl-prolyl cis-trans isomerase activity | 3.87E-04 | 12 |
| metal ion transmembrane transporter activity | 4.98E-04 | 32 | GDP binding | 3.87E-04 | 12 |
| regulation of translational initiation | 4.98E-04 | 32 | RNA-directed DNA polymerase activity | 3.87E-04 | 12 |
| protein heterodimerization activity | 4.98E-04 | 32 | Ras GTPase binding | 3.87E-04 | 12 |
| protein serine/threonine kinase activity | 4.98E-04 | 32 | cullin-RING ubiquitin ligase complex | 7.97E-04 | 11 |
| interspecies interaction between organisms | 5.38E-04 | 92 | protein tyrosine kinase activity | 7.97E-04 | 11 |
| nucleotidyltransferase activity | 6.65E-04 | 31 | oxidoreductase activity, acting on single donors with incorporation of molecular oxygen, incorporation of two atoms of oxygen | 7.97E-04 | 11 |
| endopeptidase inhibitor activity | 6.65E-04 | 31 | serine-type endopeptidase inhibitor activity | 7.97E-04 | 11 |
| regulation of inflammatory response | 6.65E-04 | 31 | lipoprotein particle receptor binding | 7.97E-04 | 11 |
| gluconeogenesis | 6.65E-04 | 31 | synaptosome | 7.97E-04 | 11 |
| early endosome | 6.65E-04 | 31 | protein serine/threonine phosphatase complex | 7.97E-04 | 11 |
| basolateral plasma membrane | 6.65E-04 | 31 | 4 iron, 4 sulfur cluster binding | 7.97E-04 | 11 |
| M/G1 transition of mitotic cell cycle | 6.65E-04 | 31 | mitochondrial intermembrane space | 7.97E-04 | 11 |
| negative regulation of cell differentiation | 6.65E-04 | 31 | rough endoplasmic reticulum | 7.97E-04 | 11 |
| methyltransferase activity | 8.94E-04 | 30 | proteasome core complex | 7.97E-04 | 11 |
| insulin receptor signaling pathway | 8.94E-04 | 30 | eukaryotic translation initiation factor 3 complex | 7.97E-04 | 11 |
| regulation of cell morphogenesis | 8.94E-04 | 30 | steroid hormone receptor activity | 7.97E-04 | 11 |
| transmembrane receptor protein serine/threonine kinase signaling pathway | 8.94E-04 | 30 | focal adhesion | 7.97E-04 | 11 |
| regulation of secretion | 1.18E-03 | 29 | threonine-type endopeptidase activity | 7.97E-04 | 11 |
| DNA damage response, signal transduction by p53 class mediator resulting in cell cycle arrest | 1.18E-03 | 29 | nuclear matrix | 7.97E-04 | 11 |
| cholesterol metabolic process | 1.18E-03 | 29 | fatty-acyl-CoA binding | 7.97E-04 | 11 |
| rRNA processing | 1.18E-03 | 29 | DNA-directed RNA polymerase II, holoenzyme | 7.97E-04 | 11 |
| endosomal transport | 1.18E-03 | 29 | eukaryotic cell surface binding | 1.64E-03 | 10 |
| cell leading edge | 1.18E-03 | 29 | calcium-dependent protein binding | 1.64E-03 | 10 |
| oxidoreductase activity, acting on the CH-OH group of donors, NAD or NADP as acceptor | 1.18E-03 | 29 | NADP binding | 1.64E-03 | 10 |
| heart development | 1.57E-03 | 28 | inorganic anion transmembrane transporter activity | 1.64E-03 | 10 |
| protein C-terminus binding | 1.57E-03 | 28 | low-density lipoprotein particle | 1.64E-03 | 10 |
| embryonic morphogenesis | 1.57E-03 | 28 | spherical high-density lipoprotein particle | 1.64E-03 | 10 |
| regulation of cell adhesion | 1.57E-03 | 28 | transcription regulatory region DNA binding | 1.64E-03 | 10 |
| cytosolic large ribosomal subunit | 1.57E-03 | 28 | nuclear chromatin | 1.64E-03 | 10 |
| regulation of cellular amino acid metabolic process | 1.57E-03 | 28 | replication fork | 1.64E-03 | 10 |
| response to radiation | 1.57E-03 | 28 | integrin complex | 1.64E-03 | 10 |
| response to ethanol | 1.57E-03 | 28 | peroxisomal matrix | 1.64E-03 | 10 |
| cytokine-mediated signaling pathway | 1.57E-03 | 28 | caveola | 1.64E-03 | 10 |
| microtubule cytoskeleton organization | 1.57E-03 | 28 | translation elongation factor activity | 1.64E-03 | 10 |
| mitochondrial transport | 1.57E-03 | 28 | glutathione transferase activity | 1.64E-03 | 10 |
| amino acid transport | 1.57E-03 | 28 | chylomicron | 1.64E-03 | 10 |
| drug metabolic process | 2.08E-03 | 27 | ubiquitin binding | 1.64E-03 | 10 |
| acute-phase response | 2.08E-03 | 27 | phosphatidylinositol-4,5-bisphosphate 5-phosphatase activity | 1.64E-03 | 10 |
| response to virus | 2.08E-03 | 27 | myosin complex | 1.64E-03 | 10 |
| dicarboxylic acid metabolic process | 2.08E-03 | 27 | leading edge membrane | 1.64E-03 | 10 |
| activation of protein kinase activity | 2.08E-03 | 27 | Cajal body | 3.34E-03 | 9 |
| purine nucleobase metabolic process | 2.08E-03 | 27 | hydrolase activity, acting on carbon-nitrogen (but not peptide) bonds, in linear amides | 3.34E-03 | 9 |
| protein N-linked glycosylation via asparagine | 2.08E-03 | 27 | cysteine-type endopeptidase inhibitor activity | 3.34E-03 | 9 |
| axon | 2.08E-03 | 27 | carboxy-lyase activity | 3.34E-03 | 9 |
| transferase activity, transferring hexosyl groups | 2.76E-03 | 26 | cytokine receptor activity | 3.34E-03 | 9 |
| reactive oxygen species metabolic process | 2.76E-03 | 26 | divalent inorganic cation transmembrane transporter activity | 3.34E-03 | 9 |
| steroid binding | 2.76E-03 | 26 | phosphoprotein binding | 3.34E-03 | 9 |
| microtubule associated complex | 2.76E-03 | 26 | chemokine activity | 3.34E-03 | 9 |
| regulation of GTPase activity | 2.76E-03 | 26 | G-protein coupled receptor activity | 3.34E-03 | 9 |
| sphingolipid metabolic process | 2.76E-03 | 26 | growth factor activity | 3.34E-03 | 9 |
| liver development | 2.76E-03 | 26 | solute:cation symporter activity | 3.34E-03 | 9 |
| negative regulation of transcription from RNA polymerase II promoter | 2.76E-03 | 26 | N-methyltransferase activity | 3.34E-03 | 9 |
| ubiquitin ligase complex | 2.76E-03 | 26 | collagen binding | 3.34E-03 | 9 |
| water-soluble vitamin metabolic process | 2.76E-03 | 26 | manganese ion binding | 3.34E-03 | 9 |
| response to acid | 3.65E-03 | 25 | nuclear pore | 3.34E-03 | 9 |
| androgen metabolic process | 3.65E-03 | 25 | cholesterol binding | 3.34E-03 | 9 |
| clathrin-coated vesicle | 3.65E-03 | 25 | extrinsic to plasma membrane | 3.34E-03 | 9 |
| basement membrane | 3.65E-03 | 25 | mitochondrial outer membrane translocase complex | 3.34E-03 | 9 |
| signal release | 3.65E-03 | 25 | protein phosphatase binding | 3.34E-03 | 9 |
| lung development | 3.65E-03 | 25 | Golgi cisterna membrane | 3.34E-03 | 9 |
| regulation of transmembrane transport | 3.65E-03 | 25 | kinesin complex | 3.34E-03 | 9 |
| regulation of lipid biosynthetic process | 3.65E-03 | 25 | actin filament | 3.34E-03 | 9 |
| membrane raft | 3.65E-03 | 25 | aminopeptidase activity | 3.34E-03 | 9 |
| serine-type endopeptidase activity | 3.65E-03 | 25 | growth cone | 3.34E-03 | 9 |
| cell-cell adhesion | 3.65E-03 | 25 | midbody | 3.34E-03 | 9 |
| tryptophan metabolic process | 3.65E-03 | 25 | DNA-directed RNA polymerase activity | 3.34E-03 | 9 |
| triglyceride metabolic process | 3.65E-03 | 25 | axon part | 3.34E-03 | 9 |
| nerve growth factor receptor signaling pathway | 3.65E-03 | 25 | stress fiber | 3.34E-03 | 9 |
| receptor-mediated endocytosis | 3.65E-03 | 25 | lipase inhibitor activity | 3.34E-03 | 9 |
| cytokine receptor binding | 3.65E-03 | 25 | glycoprotein binding | 3.34E-03 | 9 |
| leukocyte differentiation | 4.81E-03 | 24 | oxidoreductase activity, acting on the aldehyde or oxo group of donors, NAD or NADP as acceptor | 3.34E-03 | 9 |
| cell surface binding | 4.81E-03 | 24 | transferase activity, transferring pentosyl groups | 6.79E-03 | 8 |
| striated muscle tissue development | 4.81E-03 | 24 | brush border membrane | 6.79E-03 | 8 |
| external side of plasma membrane | 4.81E-03 | 24 | intramolecular oxidoreductase activity, transposing C=C bonds | 6.79E-03 | 8 |
| methylation | 4.81E-03 | 24 | beta-tubulin binding | 6.79E-03 | 8 |
| cellular response to reactive oxygen species | 4.81E-03 | 24 | synaptic vesicle | 6.79E-03 | 8 |
| pyrimidine nucleobase metabolic process | 4.81E-03 | 24 | ER to Golgi transport vesicle membrane | 6.79E-03 | 8 |
| protein transporter activity | 4.81E-03 | 24 | ubiquinol-cytochrome-c reductase activity | 6.79E-03 | 8 |
| mRNA binding | 4.81E-03 | 24 | sarcomere | 6.79E-03 | 8 |
| translation initiation factor activity | 4.81E-03 | 24 | oligosaccharyltransferase complex | 6.79E-03 | 8 |
| response to hydrogen peroxide | 4.81E-03 | 24 | protein transmembrane transporter activity | 6.79E-03 | 8 |
| ribonucleoprotein complex binding | 4.81E-03 | 24 | aldo-keto reductase (NADP) activity | 6.79E-03 | 8 |
| monocarboxylic acid binding | 4.81E-03 | 24 | vitamin D3 25-hydroxylase activity | 6.79E-03 | 8 |
| actin filament organization | 4.81E-03 | 24 | proteasome regulatory particle | 6.79E-03 | 8 |
| protein processing | 4.81E-03 | 24 | polysome | 6.79E-03 | 8 |
| receptor complex | 4.81E-03 | 24 | toxin binding | 6.79E-03 | 8 |
| epithelial cell differentiation | 4.81E-03 | 24 | steroid hormone receptor binding | 6.79E-03 | 8 |
| small GTPase regulator activity | 4.81E-03 | 24 | cysteine-type endopeptidase activity | 6.79E-03 | 8 |
| carboxylic ester hydrolase activity | 4.81E-03 | 24 | hydrogen-exporting ATPase activity, phosphorylative mechanism | 6.79E-03 | 8 |
| chaperone binding | 6.40E-03 | 23 | RNA polymerase II distal enhancer sequence-specific DNA binding transcription factor activity | 6.79E-03 | 8 |
| regulation of sequence-specific DNA binding transcription factor activity | 6.40E-03 | 23 | double-stranded RNA binding | 6.79E-03 | 8 |
| regulation of binding | 6.40E-03 | 23 | postsynaptic density | 6.79E-03 | 8 |
| muscle organ development | 6.40E-03 | 23 | MHC class I protein complex | 6.79E-03 | 8 |
| positive regulation of response to external stimulus | 6.40E-03 | 23 | protein binding, bridging | 6.79E-03 | 8 |
| heparin binding | 6.40E-03 | 23 | histone deacetylase complex | 6.79E-03 | 8 |
| glycolysis | 6.40E-03 | 23 | histone acetyltransferase complex | 6.79E-03 | 8 |
| regulation of Ras protein signal transduction | 6.40E-03 | 23 | endoribonuclease activity | 6.79E-03 | 8 |
| spindle | 6.40E-03 | 23 | exonuclease activity | 6.79E-03 | 8 |
| cell-cell junction | 6.40E-03 | 23 | protein kinase C binding | 6.79E-03 | 8 |
| glycine metabolic process | 6.40E-03 | 23 | ribonucleoprotein granule | 6.79E-03 | 8 |
| cholesterol efflux | 6.40E-03 | 23 | oxidoreductase activity, acting on the CH-CH group of donors, NAD or NADP as acceptor | 6.79E-03 | 8 |
| protein import into nucleus | 6.40E-03 | 23 | integrin binding | 1.36E-02 | 7 |
| defense response to bacterium | 6.40E-03 | 23 | NF-kappaB binding | 1.36E-02 | 7 |
| terpenoid metabolic process | 6.40E-03 | 23 | structural constituent of cytoskeleton | 1.36E-02 | 7 |
| amino acid binding | 6.40E-03 | 23 | protein disulfide oxidoreductase activity | 1.36E-02 | 7 |
| magnesium ion binding | 6.40E-03 | 23 | cation channel activity | 1.36E-02 | 7 |
| regulation of neuron differentiation | 8.39E-03 | 22 | cofactor transporter activity | 1.36E-02 | 7 |
| platelet degranulation | 8.39E-03 | 22 | SMAD binding | 1.36E-02 | 7 |
| response to unfolded protein | 8.39E-03 | 22 | sterol transporter activity | 1.36E-02 | 7 |
| embryonic organ development | 8.39E-03 | 22 | COPI vesicle coat | 1.36E-02 | 7 |
| secondary active transmembrane transporter activity | 8.39E-03 | 22 | PDZ domain binding | 1.36E-02 | 7 |
| estrogen metabolic process | 8.39E-03 | 22 | intercellular canaliculus | 1.36E-02 | 7 |
| pyruvate metabolic process | 8.39E-03 | 22 | histone methyltransferase complex | 1.36E-02 | 7 |
| regulation of proteolysis | 8.39E-03 | 22 | acetylglucosaminyltransferase activity | 1.36E-02 | 7 |
| peroxisomal part | 8.39E-03 | 22 | spindle pole | 1.36E-02 | 7 |
| vesicle organization | 8.39E-03 | 22 | transaminase activity | 1.36E-02 | 7 |
| regulation of membrane potential | 8.39E-03 | 22 | integral to mitochondrial membrane | 1.36E-02 | 7 |
| glycerolipid biosynthetic process | 8.39E-03 | 22 | hemoglobin complex | 1.36E-02 | 7 |
| cell cortex | 8.39E-03 | 22 | histone binding | 1.36E-02 | 7 |
| organic anion transport | 8.39E-03 | 22 | damaged DNA binding | 1.36E-02 | 7 |
| polysaccharide metabolic process | 8.39E-03 | 22 | cytoplasmic dynein complex | 1.36E-02 | 7 |
| morphogenesis of an epithelium | 8.39E-03 | 22 | intermediate filament | 1.36E-02 | 7 |
| phospholipid biosynthetic process | 8.39E-03 | 22 | growth factor receptor binding | 1.36E-02 | 7 |
| lysine catabolic process | 8.39E-03 | 22 | organic anion transmembrane transporter activity | 1.36E-02 | 7 |
| tubulin complex | 8.39E-03 | 22 | gated channel activity | 1.36E-02 | 7 |
| L-serine metabolic process | 8.39E-03 | 22 | RNA helicase activity | 1.36E-02 | 7 |
| fatty acid beta-oxidation | 8.39E-03 | 22 | single-stranded RNA binding | 1.36E-02 | 7 |
| regulation of lipase activity | 8.39E-03 | 22 | microvillus | 1.36E-02 | 7 |
| regulation of fatty acid metabolic process | 8.39E-03 | 22 | endoplasmic reticulum-Golgi intermediate compartment membrane | 1.36E-02 | 7 |
| GTPase activator activity | 8.39E-03 | 22 | proton-transporting V-type ATPase complex | 1.36E-02 | 7 |
| regulation of epithelial cell proliferation | 1.11E-02 | 21 | proton-transporting two-sector ATPase complex, catalytic domain | 1.36E-02 | 7 |
| response to activity | 1.11E-02 | 21 | steroid dehydrogenase activity | 1.36E-02 | 7 |
| cellular aldehyde metabolic process | 1.11E-02 | 21 | hydrogen ion transporting ATP synthase activity, rotational mechanism | 1.36E-02 | 7 |
| transport vesicle | 1.11E-02 | 21 | proton-transporting ATPase activity, rotational mechanism | 1.36E-02 | 7 |
| gland development | 1.11E-02 | 21 | secretory granule membrane | 1.36E-02 | 7 |
| endoplasmic reticulum lumen | 1.11E-02 | 21 | mannosyl-oligosaccharide mannosidase activity | 1.36E-02 | 7 |
| endoplasmic reticulum-Golgi intermediate compartment | 1.11E-02 | 21 | tRNA binding | 1.36E-02 | 7 |
| centrosome | 1.11E-02 | 21 | complement binding | 1.36E-02 | 7 |
| tube morphogenesis | 1.11E-02 | 21 | dolichyl-diphosphooligosaccharide-protein glycotransferase activity | 1.36E-02 | 7 |
| protein dephosphorylation | 1.11E-02 | 21 | signal sequence binding | 1.36E-02 | 7 |
| threonine metabolic process | 1.11E-02 | 21 | phospholipase activity | 1.36E-02 | 7 |
| glutathione metabolic process | 1.11E-02 | 21 | thiamine pyrophosphate binding | 2.65E-02 | 6 |
| cellular calcium ion homeostasis | 1.11E-02 | 21 | SH3 domain binding | 2.65E-02 | 6 |
| cellular iron ion homeostasis | 1.11E-02 | 21 | hormone activity | 2.65E-02 | 6 |
| regulation of MAPK cascade | 1.46E-02 | 20 | actin filament binding | 2.65E-02 | 6 |
| phagocytosis | 1.46E-02 | 20 | receptor signaling protein serine/threonine kinase activity | 2.65E-02 | 6 |
| flavin adenine dinucleotide binding | 1.46E-02 | 20 | protein serine/threonine phosphatase activity | 2.65E-02 | 6 |
| regulation of angiogenesis | 1.46E-02 | 20 | protein tyrosine phosphatase activity | 2.65E-02 | 6 |
| female pregnancy | 1.46E-02 | 20 | oxidoreductase activity, acting on paired donors, with incorporation or reduction of molecular oxygen, 2-oxoglutarate as one donor, and incorporation of one atom each of oxygen into both donors | 2.65E-02 | 6 |
| positive regulation of lipid metabolic process | 1.46E-02 | 20 | phosphotransferase activity, phosphate group as acceptor | 2.65E-02 | 6 |
| high-density lipoprotein particle | 1.46E-02 | 20 | testosterone 6-beta-hydroxylase activity | 2.65E-02 | 6 |
| developmental growth | 1.46E-02 | 20 | methyl indole-3-acetate esterase activity | 2.65E-02 | 6 |
| myeloid cell differentiation | 1.46E-02 | 20 | methyl salicylate esterase activity | 2.65E-02 | 6 |
| hormone transport | 1.46E-02 | 20 | methyl jasmonate esterase activity | 2.65E-02 | 6 |
| bile acid metabolic process | 1.46E-02 | 20 | ATPase binding | 2.65E-02 | 6 |
| regulation of endocytosis | 1.46E-02 | 20 | ADP binding | 2.65E-02 | 6 |
| tricarboxylic acid cycle | 1.46E-02 | 20 | thyroid hormone receptor activity | 2.65E-02 | 6 |
| endocytic vesicle | 1.46E-02 | 20 | racemase and epimerase activity, acting on carbohydrates and derivatives | 2.65E-02 | 6 |
| response to estradiol stimulus | 1.46E-02 | 20 | intramolecular transferase activity | 2.65E-02 | 6 |
| regulation of cholesterol transport | 1.46E-02 | 20 | ligase activity, forming carbon-carbon bonds | 2.65E-02 | 6 |
| regulation of protein catabolic process | 1.46E-02 | 20 | RS domain binding | 2.65E-02 | 6 |
| mitochondrial electron transport, cytochrome c to oxygen | 1.46E-02 | 20 | iron ion transmembrane transporter activity | 2.65E-02 | 6 |
| nuclear membrane | 1.46E-02 | 20 | L-serine transmembrane transporter activity | 2.65E-02 | 6 |
| synapse part | 1.46E-02 | 20 | cleavage furrow | 2.65E-02 | 6 |
| lysosomal membrane | 1.46E-02 | 20 | intermediate-density lipoprotein particle | 2.65E-02 | 6 |
| Golgi stack | 1.46E-02 | 20 | extracellular matrix binding | 2.65E-02 | 6 |
| gonad development | 1.46E-02 | 20 | drug transmembrane transporter activity | 2.65E-02 | 6 |
| anion transmembrane transporter activity | 1.46E-02 | 20 | antiporter activity | 2.65E-02 | 6 |
| skeletal system development | 1.46E-02 | 20 | ferric iron binding | 2.65E-02 | 6 |
| organelle assembly | 1.46E-02 | 20 | basal plasma membrane | 2.65E-02 | 6 |
| G-protein coupled receptor binding | 1.46E-02 | 20 | membrane attack complex | 2.65E-02 | 6 |
| isoleucine metabolic process | 1.46E-02 | 20 | vitamin D 24-hydroxylase activity | 2.65E-02 | 6 |
| valine metabolic process | 1.46E-02 | 20 | condensed chromosome kinetochore | 2.65E-02 | 6 |
| regulation of ion transport | 1.46E-02 | 20 | clathrin vesicle coat | 2.65E-02 | 6 |
| response to heat | 1.46E-02 | 20 | trans-Golgi network transport vesicle | 2.65E-02 | 6 |
| forebrain development | 1.46E-02 | 20 | integral to Golgi membrane | 2.65E-02 | 6 |
| mRNA transport | 1.92E-02 | 19 | isoprenoid binding | 2.65E-02 | 6 |
| 'de novo' posttranslational protein folding | 1.92E-02 | 19 | protein phosphatase regulator activity | 2.65E-02 | 6 |
| complement activation | 1.92E-02 | 19 | autophagic vacuole | 2.65E-02 | 6 |
| striated muscle cell differentiation | 1.92E-02 | 19 | integral to peroxisomal membrane | 2.65E-02 | 6 |
| protein polymerization | 1.92E-02 | 19 | ATP-dependent DNA helicase activity | 2.65E-02 | 6 |
| protein tetramerization | 1.92E-02 | 19 | cytokine binding | 2.65E-02 | 6 |
| regulation of T cell activation | 1.92E-02 | 19 | biotin carboxylase activity | 2.65E-02 | 6 |
| sulfotransferase activity | 1.92E-02 | 19 | sarcolemma | 2.65E-02 | 6 |
| regulation of reproductive process | 1.92E-02 | 19 | ATP-dependent peptidase activity | 2.65E-02 | 6 |
| phosphatidylinositol metabolic process | 1.92E-02 | 19 | beta-amyloid binding | 2.65E-02 | 6 |
| phosphatidylinositol binding | 1.92E-02 | 19 | caffeine oxidase activity | 2.65E-02 | 6 |
| cellular response to lipopolysaccharide | 1.92E-02 | 19 | receptor inhibitor activity | 2.65E-02 | 6 |
| cellular response to vitamin | 1.92E-02 | 19 | postsynaptic membrane | 2.65E-02 | 6 |
| divalent metal ion transport | 1.92E-02 | 19 | oxoglutarate dehydrogenase complex | 2.65E-02 | 6 |
| cytochrome-c oxidase activity | 1.92E-02 | 19 | recycling endosome membrane | 2.65E-02 | 6 |
| nucleosome assembly | 1.92E-02 | 19 | protein N-terminus binding | 2.65E-02 | 6 |
| transcription corepressor activity | 1.92E-02 | 19 | clathrin-coated endocytic vesicle membrane | 2.65E-02 | 6 |
| coated pit | 1.92E-02 | 19 | biotin carboxylase complex | 2.65E-02 | 6 |
| glutamine family amino acid metabolic process | 1.92E-02 | 19 | anchored to membrane | 2.65E-02 | 6 |
| branched chain family amino acid catabolic process | 1.92E-02 | 19 | biotin binding | 2.65E-02 | 6 |
| protease binding | 1.92E-02 | 19 | perikaryon | 2.65E-02 | 6 |
| response to vitamin D | 1.92E-02 | 19 | transmembrane receptor protein kinase activity | 2.65E-02 | 6 |
| respiratory chain complex IV | 1.92E-02 | 19 | nuclear ubiquitin ligase complex | 2.65E-02 | 6 |
| regulation of homeostatic process | 1.92E-02 | 19 | cytoplasmic ubiquitin ligase complex | 2.65E-02 | 6 |
| cholesterol homeostasis | 1.92E-02 | 19 | phospholipase binding | 2.65E-02 | 6 |
| positive regulation of cytokine production | 1.92E-02 | 19 | extracellular membrane-bounded organelle | 2.65E-02 | 6 |
| cell projection membrane | 1.92E-02 | 19 | peptidase activator activity | 2.65E-02 | 6 |
| regulation of protein complex assembly | 1.92E-02 | 19 | glutathione binding | 2.65E-02 | 6 |
| cell redox homeostasis | 1.92E-02 | 19 | cortical actin cytoskeleton | 2.65E-02 | 6 |
| regulation of cell projection organization | 1.92E-02 | 19 | scavenger receptor activity | 2.65E-02 | 6 |
| protein polyubiquitination | 1.92E-02 | 19 | carbohydrate kinase activity | 2.65E-02 | 6 |
| pattern specification process | 2.53E-02 | 18 | Ras guanyl-nucleotide exchange factor activity | 2.65E-02 | 6 |
| positive regulation of MAP kinase activity | 2.53E-02 | 18 | peptide hormone binding | 2.65E-02 | 6 |
| positive regulation of immune effector process | 2.53E-02 | 18 | translation initiation factor binding | 2.65E-02 | 6 |
| response to carbohydrate stimulus | 2.53E-02 | 18 | glutathione peroxidase activity | 2.65E-02 | 6 |
| regulation of cellular response to stress | 2.53E-02 | 18 | mitochondrial proton-transporting ATP synthase complex, coupling factor F(o) | 2.65E-02 | 6 |
| ubiquitin protein ligase binding | 2.53E-02 | 18 | oxidoreductase activity, acting on the CH-NH group of donors, NAD or NADP as acceptor | 2.65E-02 | 6 |
| response to interferon-gamma | 2.53E-02 | 18 |  |  |  |
| regulation of synaptic transmission | 2.53E-02 | 18 |  |  |  |
| positive regulation of behavior | 2.53E-02 | 18 |  |  |  |
| circadian rhythm | 2.53E-02 | 18 |  |  |  |
| nicotinamide nucleotide metabolic process | 2.53E-02 | 18 |  |  |  |
| regulation of Wnt receptor signaling pathway | 2.53E-02 | 18 |  |  |  |
| mitochondrial large ribosomal subunit | 2.53E-02 | 18 |  |  |  |
| negative regulation of cell growth | 2.53E-02 | 18 |  |  |  |
| regulation of cellular component size | 2.53E-02 | 18 |  |  |  |
| DNA-dependent transcription, termination | 2.53E-02 | 18 |  |  |  |
| melanosome | 2.53E-02 | 18 |  |  |  |
| monocarboxylic acid transport | 2.53E-02 | 18 |  |  |  |
| response to amine stimulus | 2.53E-02 | 18 |  |  |  |
| leukocyte chemotaxis | 2.53E-02 | 18 |  |  |  |
| amine transport | 2.53E-02 | 18 |  |  |  |
| platelet alpha granule | 2.53E-02 | 18 |  |  |  |
| leucine metabolic process | 2.53E-02 | 18 |  |  |  |
| microtubule-based movement | 2.53E-02 | 18 |  |  |  |
| morphogenesis of a branching structure | 2.53E-02 | 18 |  |  |  |
| positive regulation of I-kappaB kinase/NF-kappaB cascade | 2.53E-02 | 18 |  |  |  |
| protein-DNA complex | 2.53E-02 | 18 |  |  |  |
| epidermal growth factor receptor signaling pathway | 2.53E-02 | 18 |  |  |  |
| cell killing | 2.53E-02 | 18 |  |  |  |
| regulation of DNA metabolic process | 3.31E-02 | 17 |  |  |  |
| hydrolase activity, acting on glycosyl bonds | 3.31E-02 | 17 |  |  |  |
| negative regulation of neuron apoptotic process | 3.31E-02 | 17 |  |  |  |
| amino acid transmembrane transporter activity | 3.31E-02 | 17 |  |  |  |
| regulation of viral reproduction | 3.31E-02 | 17 |  |  |  |
| microtubule binding | 3.31E-02 | 17 |  |  |  |
| positive regulation of lymphocyte activation | 3.31E-02 | 17 |  |  |  |
| negative regulation of lipid metabolic process | 3.31E-02 | 17 |  |  |  |
| locomotory behavior | 3.31E-02 | 17 |  |  |  |
| regulation of blood vessel size | 3.31E-02 | 17 |  |  |  |
| calmodulin binding | 3.31E-02 | 17 |  |  |  |
| vesicle coat | 3.31E-02 | 17 |  |  |  |
| pyridoxal phosphate binding | 3.31E-02 | 17 |  |  |  |
| rRNA binding | 3.31E-02 | 17 |  |  |  |
| iron-sulfur cluster binding | 3.31E-02 | 17 |  |  |  |
| peptidyl-tyrosine phosphorylation | 3.31E-02 | 17 |  |  |  |
| negative regulation of organelle organization | 3.31E-02 | 17 |  |  |  |
| nuclear chromosome part | 3.31E-02 | 17 |  |  |  |
| Golgi-associated vesicle | 3.31E-02 | 17 |  |  |  |
| maintenance of location in cell | 3.31E-02 | 17 |  |  |  |
| sequence-specific DNA binding RNA polymerase II transcription factor activity | 3.31E-02 | 17 |  |  |  |
| immunoglobulin mediated immune response | 3.31E-02 | 17 |  |  |  |
| DNA recombination | 3.31E-02 | 17 |  |  |  |
| positive regulation of innate immune response | 3.31E-02 | 17 |  |  |  |
| epidermis development | 3.31E-02 | 17 |  |  |  |
| RNA export from nucleus | 3.31E-02 | 17 |  |  |  |
| intracellular steroid hormone receptor signaling pathway | 3.31E-02 | 17 |  |  |  |
| small nuclear ribonucleoprotein complex | 3.31E-02 | 17 |  |  |  |
| response to starvation | 3.31E-02 | 17 |  |  |  |
| proton-transporting ATP synthase complex | 3.31E-02 | 17 |  |  |  |
| tyrosine metabolic process | 3.31E-02 | 17 |  |  |  |
| ATPase activity, coupled to transmembrane movement of ions | 3.31E-02 | 17 |  |  |  |
| acetyltransferase activity | 3.31E-02 | 17 |  |  |  |
| negative regulation of cytokine production | 3.31E-02 | 17 |  |  |  |
| regulation of actin cytoskeleton organization | 3.31E-02 | 17 |  |  |  |
| intrinsic to endoplasmic reticulum membrane | 3.31E-02 | 17 |  |  |  |
| histone modification | 3.31E-02 | 17 |  |  |  |
| negative regulation of protein phosphorylation | 3.31E-02 | 17 |  |  |  |
| negative regulation of cellular catabolic process | 3.31E-02 | 17 |  |  |  |
| iron ion transport | 3.31E-02 | 17 |  |  |  |
| post-Golgi vesicle-mediated transport | 3.31E-02 | 17 |  |  |  |
| anchoring junction | 4.32E-02 | 16 |  |  |  |
| autophagy | 4.32E-02 | 16 |  |  |  |
| muscle contraction | 4.32E-02 | 16 |  |  |  |
| cell-substrate adhesion | 4.32E-02 | 16 |  |  |  |
| hydro-lyase activity | 4.32E-02 | 16 |  |  |  |
| lipid transporter activity | 4.32E-02 | 16 |  |  |  |
| aromatase activity | 4.32E-02 | 16 |  |  |  |
| positive regulation of protein transport | 4.32E-02 | 16 |  |  |  |
| immune response-activating signal transduction | 4.32E-02 | 16 |  |  |  |
| cell junction organization | 4.32E-02 | 16 |  |  |  |
| plasma lipoprotein particle clearance | 4.32E-02 | 16 |  |  |  |
| regulation of coagulation | 4.32E-02 | 16 |  |  |  |
| establishment of protein localization in mitochondrion | 4.32E-02 | 16 |  |  |  |
| gliogenesis | 4.32E-02 | 16 |  |  |  |
| hormone receptor binding | 4.32E-02 | 16 |  |  |  |
| regulation of blood pressure | 4.32E-02 | 16 |  |  |  |
| extracellular matrix organization | 4.32E-02 | 16 |  |  |  |
| immunoglobulin complex | 4.32E-02 | 16 |  |  |  |
| leukocyte proliferation | 4.32E-02 | 16 |  |  |  |
| energy reserve metabolic process | 4.32E-02 | 16 |  |  |  |
| organelle subcompartment | 4.32E-02 | 16 |  |  |  |
| establishment of vesicle localization | 4.32E-02 | 16 |  |  |  |
| trans-Golgi network | 4.32E-02 | 16 |  |  |  |
| multicellular organism growth | 4.32E-02 | 16 |  |  |  |
| cellular response to growth factor stimulus | 4.32E-02 | 16 |  |  |  |
| cellular response to steroid hormone stimulus | 4.32E-02 | 16 |  |  |  |
| glucose transport | 4.32E-02 | 16 |  |  |  |
| metalloendopeptidase activity | 4.32E-02 | 16 |  |  |  |
| tRNA aminoacylation for protein translation | 4.32E-02 | 16 |  |  |  |
| regulation of glucose metabolic process | 4.32E-02 | 16 |  |  |  |
| induction of apoptosis by intracellular signals | 4.32E-02 | 16 |  |  |  |
| urogenital system development | 4.32E-02 | 16 |  |  |  |
| camera-type eye development | 4.32E-02 | 16 |  |  |  |
| heat shock protein binding | 4.32E-02 | 16 |  |  |  |
| sulfation | 4.32E-02 | 16 |  |  |  |
| helicase activity | 4.32E-02 | 16 |  |  |  |
| organ regeneration | 4.32E-02 | 16 |  |  |  |
| ATP synthesis coupled proton transport | 4.32E-02 | 16 |  |  |  |
| protein targeting to membrane | 4.32E-02 | 16 |  |  |  |
| N-acyltransferase activity | 4.32E-02 | 16 |  |  |  |
| glycolipid metabolic process | 4.32E-02 | 16 |  |  |  |
| protein secretion | 4.32E-02 | 16 |  |  |  |
| fat cell differentiation | 4.32E-02 | 16 |  |  |  |
| positive regulation of cysteine-type endopeptidase activity involved in apoptotic process | 4.32E-02 | 16 |  |  |  |
| contractile fiber | 4.32E-02 | 16 |  |  |  |
| fat-soluble vitamin metabolic process | 4.32E-02 | 16 |  |  |  |
| regulation of steroid metabolic process | 4.32E-02 | 16 |  |  |  |
| Rho protein signal transduction | 4.32E-02 | 16 |  |  |  |
| positive regulation of cellular catabolic process | 4.32E-02 | 16 |  |  |  |
| cytokine activity | 4.32E-02 | 16 |  |  |  |
| mitochondrion organization | 4.97E-02 | 84 |  |  |  |

**B**

| Brain to Liver | | | Brain to Gonads | | |
| --- | --- | --- | --- | --- | --- |
| GO term | FDR | # Sequences | GO term | FDR | # Sequences |
| plasma membrane | 2.41E-44 | 567 | plasma membrane | 4.30E-92 | 567 |
| cell-cell signaling | 3.86E-40 | 232 | cell-cell signaling | 2.79E-61 | 232 |
| cell differentiation | 1.11E-38 | 372 | signal transduction | 2.15E-39 | 525 |
| cytoskeleton organization | 1.48E-24 | 145 | cytoskeleton | 9.39E-34 | 369 |
| multicellular organismal development | 2.94E-24 | 555 | anatomical structure morphogenesis | 1.89E-30 | 291 |
| signal transduction | 1.62E-23 | 525 | cell differentiation | 5.07E-28 | 372 |
| anatomical structure morphogenesis | 1.77E-19 | 291 | behavior | 6.72E-28 | 133 |
| actin binding | 1.77E-19 | 93 | cytoplasmic membrane-bounded vesicle | 1.33E-22 | 197 |
| behavior | 2.81E-15 | 133 | ion transport | 3.13E-17 | 209 |
| ion channel activity | 6.50E-14 | 62 | ion channel activity | 5.21E-17 | 62 |
| cytoplasmic membrane-bounded vesicle | 1.72E-11 | 197 | cellular homeostasis | 9.82E-16 | 134 |
| motor activity | 2.08E-11 | 48 | actin binding | 3.54E-15 | 93 |
| protein kinase activity | 5.16E-09 | 77 | response to external stimulus | 9.26E-15 | 201 |
| protein complex | 6.06E-09 | 569 | receptor activity | 1.97E-14 | 110 |
| cellular homeostasis | 1.52E-08 | 134 | receptor binding | 2.79E-14 | 151 |
| calcium ion binding | 3.14E-07 | 109 | calcium ion binding | 7.69E-13 | 109 |
| cilium | 8.46E-07 | 27 | cytoskeleton organization | 9.13E-13 | 145 |
| ion transport | 4.08E-06 | 209 | enzyme regulator activity | 1.28E-11 | 149 |
| structural molecule activity | 1.93E-05 | 141 | Golgi apparatus | 1.51E-10 | 168 |
| transcription regulator activity | 2.73E-05 | 59 | cytosol | 1.79E-08 | 411 |
| cell cycle | 6.17E-05 | 166 | response to abiotic stimulus | 4.97E-08 | 88 |
| cytosol | 9.77E-05 | 411 | lipid binding | 5.13E-07 | 89 |
| chromosome | 1.20E-03 | 91 | response to stress | 1.13E-06 | 308 |
| microtubule organizing center | 2.08E-03 | 52 | protein complex | 1.27E-06 | 569 |
| nucleus | 2.09E-03 | 514 | cell death | 1.78E-06 | 247 |
| cell death | 3.06E-03 | 247 | motor activity | 8.38E-06 | 48 |
| response to abiotic stimulus | 3.12E-03 | 88 | endosome | 8.46E-06 | 70 |
| chromatin binding | 2.90E-02 | 29 | response to endogenous stimulus | 1.04E-05 | 122 |
| Golgi apparatus | 3.96E-02 | 168 | embryo development | 5.12E-05 | 92 |
|  |  |  | proteinaceous extracellular matrix | 1.90E-04 | 34 |
|  |  |  | nucleotide binding | 1.92E-04 | 331 |
|  |  |  | protein transport | 3.24E-04 | 152 |
|  |  |  | signal transducer activity | 8.24E-04 | 48 |
|  |  |  | protein kinase activity | 1.61E-03 | 77 |
|  |  |  | cell envelope | 1.88E-03 | 6 |
|  |  |  | carbohydrate binding | 8.60E-03 | 43 |
|  |  |  | external encapsulating structure | 1.38E-02 | 6 |
|  |  |  | structural molecule activity | 2.03E-02 | 141 |
|  |  |  | neurotransmitter transporter activity | 2.73E-02 | 6 |
|  |  |  | extracellular space | 3.68E-02 | 68 |
|  |  |  | generation of precursor metabolites and energy | 4.94E-02 | 134 |

**C**

| Gonads to Liver | | | Gonads to Brain | | |
| --- | --- | --- | --- | --- | --- |
| GO term | FDR | # Sequences | GO term | FDR | # Sequences |
| cilium | 1.26E-14 | 104 | translation | 3.96E-05 | 449 |
| cell cycle | 8.88E-12 | 571 | reproduction | 3.96E-05 | 680 |
| transcription regulator activity | 3.57E-08 | 183 | ribosome | 1.43E-03 | 383 |
| nucleolus | 2.16E-07 | 498 | viral reproduction | 4.24E-03 | 339 |
| reproduction | 1.92E-06 | 680 | nuclear chromosome | 4.34E-03 | 119 |
| nuclear chromosome | 9.86E-06 | 119 | peptidase activity | 4.51E-03 | 230 |
| cytoskeleton organization | 1.75E-04 | 218 | RNA binding | 8.05E-03 | 637 |
| nucleoplasm | 1.77E-04 | 687 | mitochondrion | 1.03E-02 | 836 |
| regulation of gene expression, epigenetic | 1.91E-04 | 41 | electron carrier activity | 1.31E-02 | 109 |
| microtubule organizing center | 6.18E-04 | 150 |  |  |  |
| protein kinase activity | 6.52E-04 | 161 |  |  |  |
| RNA binding | 6.70E-04 | 637 |  |  |  |
| structural molecule activity | 1.13E-02 | 369 |  |  |  |
| motor activity | 1.71E-02 | 64 |  |  |  |
| viral reproduction | 1.75E-02 | 339 |  |  |  |
| cell differentiation | 3.89E-02 | 613 |  |  |  |
| chromatin binding | 4.61E-02 | 78 |  |  |  |
