# Supplementary material for: Signatures of Rapid Evolution in Urban and Rural Transcriptomes of White-Footed Mice (Peromyscus leucopus) in the New York Metropolitan Area
Source: PLoS One. 2013 Aug 28;8(8):e74938. doi: 10.1371/journal.pone.0074938 (PMC3756007; doi:10.1371/journal.pone.0074938)
Supplement: Table S3 — Candidate loci with pN ⁄ pS between 0.5 and 1. (DOCX) [file pone.0074938.s005.docx]

|  | Sequence name | *p*_N_⁄*p*_S_ | Gene name | Gene function |
| --- | --- | --- | --- | --- |
| Pairwise Urban:Rural Comparisons |  |  |  |  |
|  | HP_contig02521 | 0.51 | Apoptogenic protein mitochondrial | Regulation of mitochondrial induced apoptosis |
|  | HP_contig01711 | 0.51 | Fibrinogen alpha chain | Glycoprotein circulating in the blood; functions in blood coagulation and part of the most abundant component of blood clots |
|  | HP_contig02065 | 0.52 | Complement factor h | Glycoprotein circulating in plasma; regulation of complement activation |
|  | HP_contig00430 | 0.53 | Murinoglobulin-1 precursor | Protease activity; acute phase response |
|  | HP_contig01727 | 0.54 | Ornithine mitochondrial | Transfer of ornithine across inner mitochondrial membrane |
|  | HP_contig00783 | 0.54 | Carboxymethylenebutenolidase homolog | Cysteine hydrolase, protein binding |
|  | HP_contig00807^*^ | 0.54 | Isoform cra_a | Uncharacterized cellular membrane protein |
|  | HP_contig01783 | 0.58 | Cytochrome p450 2a15 | Metabolic process; testosterone 7a-hydroxylase activity |
|  | HP_contig00737 | 0.58 | Catechol o-methyltransferase | Catalyzes methylation for degradation of neurotransmitters and catecholic xenobiotics |
|  | HP_contig05051 | 0.60 | Hypothetical protein I79_019498 | Uncharacterized |
|  | HP_contig02710 | 0.63 | L-xylulose reductase | Metabolic processes; catalyzes NADPH-dependent reduction |
|  | HP_contig01169 | 0.64 | Alpha-1-acid glycoprotein precursor | Transport protein in the blood stream; binds and distributes synthetic drugs throughout body; modulates innate immune response |
|  | HP_contig02231 | 0.66 | Isoform cra_b | Transmembrane transport protein |
|  | HP_contig02824 | 0.67 | Nadh dehydrogenase | Mitochondrial respiratory chain complex; electron transport |
|  | HP_contig03468 | 0.73 | Coatomer subunit beta | Protein transportation between Golgi body and ER; required for budding from Golgi body |
|  | HP_contig01714 | 0.74 | Complement factor i | Serine protease; regulation of complement activation by cleavage of complement system components |
|  | HP_contig01785 | 0.77 | Kininogen-1 isoform 2 precursor | Inflammatory response; involved in blood coagulation - negative regulation |
|  | HP_contig03812 | 0.79 | Biorientation of chromosomes in cell division protein 1 | Required for proper orientation of chromosomes during cell division |
|  | HP_contig01991 | 0.80 | Calcium binding and coiled-coil domain 2 | Innate immune response; receptor protein for bacteria; mediate macroautophagy |
|  | HP_contig01688 | 0.83 | Polymeric immunoglobulin receptor | Transports immunoglobulins across cell to apical surface for secretion; adaptive immune system |
|  | HP_contig02433 | 0.99 | 40s ribosomal protein s3a | RNA binding; translational initiation |
|  | HP_contig03921 | 1.00 | Bile salt sulfotransferase-like | Transferase activity |
| Pairwise Urban:Urban Comparisons |  |  |  |  |
|  | RR_contig00497 | 0.50 | Arginase-1 like | Arginine metabolism |
|  | RR_contig00554 | 0.51 | Ornithine mitochondrial | Metabolic processes; mitochondrial membrane |
|  | NYBG_contig00478^*^ | 0.54 | Fibrinogen alpha chain | Glycoprotein circulating in the blood; functions in blood coagulation and part of the most abundant component of blood clots |
|  | NYBG_contig00650 | 0.54 | Leucine-rich repeat and wd repeat-containing protein 1 | Involved with DNA replication initiation and silencing; Binds to methylated histones and restricts transcription |
|  | CP_contig00326 | 0.55 | ---NA--- | Uncharacterized |
|  | CP_contig00764 | 0.55 | Murinoglobulin-2 precursor | Protease activity; acute phase response |
|  | RR_contig00859 | 0.59 | Liver carboxylesterase b-1-like | Xenobiotic metabolism, detoxification through hydrolysis of ester and amide bonds |
|  | NYBG_contig00447 | 0.59 | Complement c3 precursor | Innate immune response; major role in activation of complement pathway |
|  | RR_contig00239 | 0.59 | Cytochrome p450 family 2 subfamily b | Xenobiotic metabolism |
|  | RR_contig00587 | 0.63 | Estradiol 17-beta-dehydrogenase 2 | Steroid biosynthetic process; oxidation-reduction |
|  | CP_contig00527 | 0.65 | Cytochrome p450 2d27-like | Xenobiotic metabolism; oxidation-reduction |
|  | NYBG_contig00664^*^ | 0.73 | Protein maelstrom homolog isoform 1 | Reproductive process; active in spermatogenesis; repress transposable elements to retain germline integrity |
|  | NYBG_contig00513 | 0.74 | Cytochrome family subfamily polypeptide 13 | Xenobiotic metabolism; oxidation-reduction |
|  | NYBG_contig00284 | 0.81 | Catechol o-methyltransferase | Methylation activity; introduces methyl groups to inactivate neurotransmitters |
|  | CP_contig00544 | 0.91 | Gametogenetin-binding protein 1 | Reproductive processes; mitochondrial morphogenesis during spermatogenesis |
|  | RR_contig01212 | 0.91 | Isoform cra_a | Uncharacterized cellular membrane protein |
|  | NYBG_contig00790^*^ | 0.98 | Orosomucoid 1 | Transport protein in the blood stream; binds and distributes synthetic drugs throughout body; modulates innate immune response |
|  | CP_contig00444^*^ | 0.99 | Vitamin d-binding protein | Albumin gene family; transports vitamin D throughout body |
|  | CP_contig00449 | 0.99 | Kininogen-1 isoform 2 precursor | Inflammatory response; involved in blood coagulation - negative regulation |

**^*^** = Gene contained *p*_N_ ⁄ *p*_S_ between 0.5 and 1 in two independent population pairwise comparisons
